# Simple dihydropyridine-based colorimetric chemosensors for heavy metal ion detection, biological evaluation, molecular docking, and ADMET profiling

**DOI:** 10.1038/s41598-023-42137-7

**Published:** 2023-09-18

**Authors:** Wafaa M. Hamada, Marwa N. El-Nahass, Ahmed A. Noser, Tarek A. Fayed, Maged El-Kemary, Maha M. Salem, Eman A. Bakr

**Affiliations:** 1 Chemistry Department, Faculty of Science, Tanta University, Tanta, 31527 Egypt; 2 Institute of Nanoscience and Nanotechnology, Kafrelsheikh University, Kafr El-Sheikh, 33516 Egypt; 3 Biochemistry Division, Chemistry Department, Faculty of Science, Tanta University, Tanta, 31527 Egypt

**Keywords:** Biochemistry, Chemistry

## Abstract

In this study, two novel chemosensors containing dihydropyridine fragment namely; (2*E*, 2*E*ʹ)-1,1ʹ-(2,6-dimethyl-1,4-dihydropyridine-3,5-diyl)bis(3-(4-(dimethylamino)phenyl)prop-2-en-1-one) **(1)**, (2*E*,2*E'*,4*E*,4*E'*)-1,1ʹ -(2,6-dimethyl-1,4-dihydropyridine-3,5-diyl)bis(5-(4-(dimethylamino)phenyl)penta-2,4-dien-1-one) **(2)** have been synthesized and characterized. The solvatochromic behavior was explored in different solvents of various polarities. The visual detection, as well as UV–Vis and fluorescence measurements were carried out to explore the colorimetric and optical sensing properties of the investigated chemosensors towards various metal ions such as Al^3+^, Cr^3+^, Mn^2+^, Fe^3+^, Co^2+^, Ni^2+^, Cu^2+^, Mg^2+^, Hg^2+^ and Zn^2+^. The chemosensors **1** and **2** have strong detecting abilities, with excellent sensitivity and selectivity for Cu^2+^ and Fe^3+^, respectively, over the other metal ions. The chemosensors were totally reversible upon addition of EDTA to the formed complexes and displayed a turn on–off-on fluorescence response based on an effect of chelation-quenching fluorescence. The antioxidant activities of the investigated chemosensors were assessed. They were examined in-silico for their capacity to block the Akt signaling pathway, which is involved in cancer proliferation with interpreting their pharmacokinetics aspects. Furthermore, in-vitro antitumor evaluation against a panel of cancer cell lines for the investigated chemosensors has been examined. Conclusively, chemosensor **1** was more effective at scavenging free radicals and as an anticancer agent and could be exploited as a therapeutic candidate for cancer therapy than chemosensor **2** due to its potential inhibition of Akt protein.

## Introduction

Optical chemosensors for harmful heavy metals detection have received more attention than other analytical methodologies due to their simplicity, great selectivity, rapid responsiveness, low cost, and straightforward usage^1–3^. Colorimetric and fluorescence chemosensors, among other optical sensors, have been employed in the detection of heavy metals in waste and biological sample analysis. They are particularly appealing because colorimetric techniques offer the convenience of visual detection of metal ions without the need of equipment, as well as precise qualitative and quantitative data that does not necessitate extensive instrumental analysis. Chalcones are a viable choice for the construction of many types of colorimetric and fluorescent chemosensors due to their visual excitation, emission, and high cell permeability^4–6^. They are the simplest aromatic ketone in the flavonoid family and may be found in natural sources such as vegetables, tea, fruits, soy foods, and spices. They have outstanding molar absorption coefficients and solvatochromic characteristics. Chalcones’ non-toxicity is another important property in comparison to other dyes; hence, chalcones are recognised as promising candidates for the continued progress of fluorescence/colorimetric probes in the chemical sensor sector^7–15^. The chalcones’ great sensitivity to metal ion concentrations allows them to examine micro-parameters of a biological entity that are connected to the polarity of the probe’s surroundings. Furthermore, chalcones have been the subject of much investigation due to their biological activity as an antidiabetic, anti-inflammatory, antioxidant, anticancer, antibacterial, anti-infective, and antipyretic. They can also be used to treat cardiovascular illness, viral disorders, parasite infections, and instances of gastritis, as well as as a means of relieving pain. The dihydropyridine moiety has good pharmacological properties such as antihypertensive, anti-inflammatory, antibacterial, antithyroidic, antiviral, antiparasitic, anti-cancer, antimuscarinic, antidiabetic, antifungal, and hypolipidemic properties^16,17^.

In light of these criteria, our study concentrated on developing simple chemosensors incorporating a dihydropyridine moiety. These were studied utilising FT-IR, ^1^H NMR, ^13^C NMR, elemental analysis, and UV–Visible studies. As a result, the application of the chemosensors to colorimetric, selective, and sensing response towards various metal ions such as Al^3+^, Cr^3+^, Mn^2+^, Fe^3+^, Co^2+^, Ni^2+^, and Cu^2+^ in ethanolic solution was also examined. Finally, the antioxidant, anticancer, and pharmacokinetic characteristics of the chemosensors were studied in-silico and in-vitro.

## Experimental section

### Materials and reagents

All chemicals were purchased from Sigma-Aldrich Chemical Co. (St. Louis, MO, USA) and used in their original form. These chemicals are acetylacetone, formaldehyde, ammonium acetate, 4-(N,N-dimethylamino)benzaldehyde, 4-(N,N-dimethyl amino)cinnamaldehyde, and urea. The investigated metal salts are AlCl_3_.6H_2_O, Cr_2_(SO_4_)_3_.15H_2_O, MnCl_2_.4H_2_O, FeCl_3_.9H_2_O, CoCl_2_·6H_2_O, NiCl_2_·6H_2_O, CuCl_2_·2H_2_O, MgCl_2_·6H_2_O, HgCl_2_ and ZnCl_2_. Methanol (MeOH), ethanol (EtOH), butanol (BuOH), iso-propanol (Iso-PrOH), acetonitrile (ACN), dichloromethane (CH_2_Cl_2_), chloroform (CHCl_3_), acetone, dimethylformamide (DMF), benzene (Benz), hexane (Hex), and toluene are the solvents used.

### Instrumentation

TLC was used to monitor reactions on precoated plates Merck Kieselgel 60 F254 (EMD Millipore Company, Billerica, MA, USA). The FT-IR spectra were recorded on a JASCO FT/IR-4100 spectrophotometer using KBr Pellets in the 4000 to 400 cm^–1^ range, and the spectra were processed using the KBr disc technique. The ^1^H NMR spectra (400 MHz) and ^13^C NMR spectra (101 MHz) were acquired at 25 °C in DMSO-d^6^ utilizing tetramethylsilane as an internal standard on a JEOL spectrometer. The elements analysis was conducted with a Perkin-Elmer 240 CHN elements analyzer. Agilent Cary Eclipse UV–Vis Scanning Spectrophotometer and Agilent Cary Eclipse Fluorescence Spectrophotometer were implemented for electronic absorption and emission spectra measurements, respectively. The fluorescence quantum yields of the examined chemosensors were determined in comparison to ethanolic Rhodamine 6G solutions (Ф_f_ = 0.95)^18^. To avoid re-absorption effects, solutions with optical densities less than 0.2 at the excitation wavelength were utilized. The picosecond fluorescence decay profiles were obtained using a FluoTime 300 (PicoQuant, Germany), and the Time-correlated single-photon counting technique. Lifetimes were computed using the FluoFit program, which was connected to the equipment.

### Synthesis of 1, 1ʹ-(2,6 dimethyl-1, 4-dihydropyridine-3,5- diyl) diethanone

The starting compound was synthesized with an 87% yield, as described by Wang et al.^19^.

#### General procedure for the synthesis of chemosensors **1** and ** 2**

**Figure 1:**
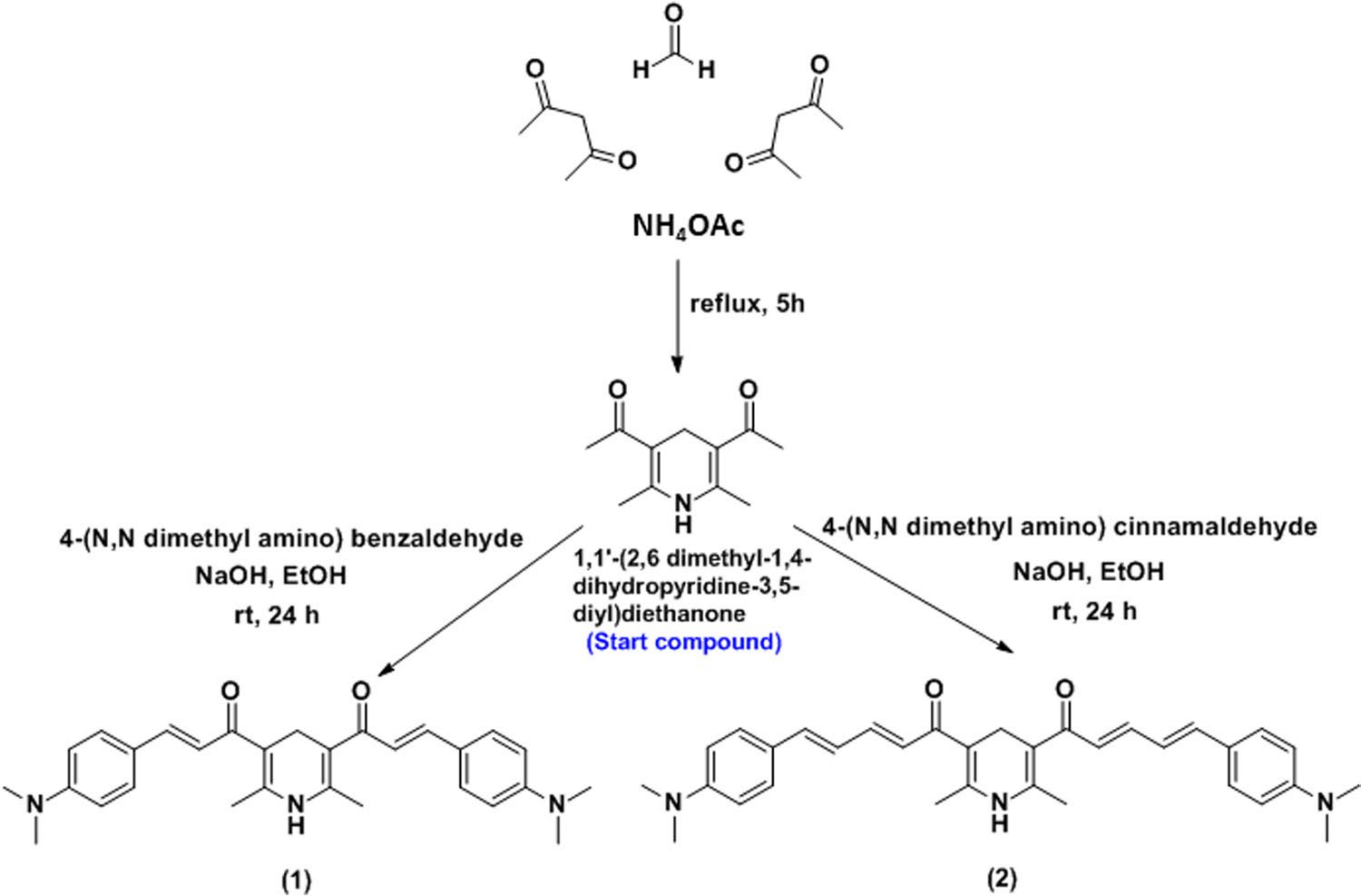
Synthesis pathway of chemosensors **1**and** 2**.

In a 100 mL conical flask, a mixture of the starting compound (4.83 g, 25 mmol), 4-(dimethylamino)benzaldehyde or 4-(dimethylamino)cinnamaldehyde (50 mmol), and sodium hydroxide (2 g, 50 mmol) in ethanol (50 mL) was stirred at room temperature for 24 h to produce chemosensor **1** and chemosensor **2**, respectively. Then, the reaction mixture was then dumped into frozen water, filtered off, and dried as described in Fig. 1.

##### (2*E*,2*E*')-1,1ʹ-(2,6-dimethyl-1,4-dihydropyridine-3,5-diyl)bis(3-(4-(dimethylamino)phenyl) prop-2-en-1-one) (1)

Orange crystals; Yield 87%; m.p. 184 °C; ^1^H NMR (400 MHz, DMSO-d^6^) δ (ppm): 9.62 (s, 1H, NH), 8.13–8.21 (d, 2H, 2CH-Ph), 7.83 (d, 2H, 2CH-CO), 6.74–7.80 (m, 8H, Ar–H), 3.80 (s, 2H, CH_2_), 2.99 (s, 12H, 2(N-(CH_3_)_2_), 2.60 (s, 6H, 2CH_3_); ^13^C NMR (101 MHz, DMSO-d^6^) δ (ppm): 190.34, 153.79, 146.41, 145.97, 126.13, 125.11, 124.49, 112.41, 111.60, 42.70, 42.20, 25.89.; IR (KBr) ν: 3443 (NH), 3077 (arom-CH), 2904 (aliph-CH), 1671 (CO), 1592 (olefinic group), 1168 (C-N).; Anal. Calcd. for C_29_H_33_N_3_O_2_ (455.59): C, 76.45%; H, 7.30%; N, 9.22% Found: C, 76.21%; H, 7.11%; N, 8.98%.

##### (2*E*,2*E*',4*E*,4*E*')-1,1ʹ-(2,6-dimethyl-1,4-dihydropyridine-3,5-diyl)bis(5-(4-(dimethylamino) phenyl)penta-2,4-dien-1-one) (2)

Reddish brown crystals; Yield 88%; m.p. 118 °C; ^1^H NMR (400 MHz, DMSO-d^6^) δ (ppm): 9.50 (s, 1H, NH), 8.19 (t, 2H, 2CH=CH–CO), 7.78 (t, 2H, 2CH=CH–Ph), 7.52 (d, 2H, 2CH–CO), 7.46 (d, 2H, 2CH–Ph), 6.68–7.44 (m, 8H, Ar–H), 3.53 (s, 2H, CH_2_), 2.92 (s, 12H, 2(N–(CH_3_)_2_), 2.61 (s, 6H, 2CH_3_).; ^13^C NMR (101 MHz, DMSO-d^6^) δ (ppm): 193.99, 154.73, 151.73, 147.44, 131.16, 129.79, 125.73, 124.17, 123.14, 112.51, 112.26, 43.52, 43.10, 16.10; IR (KBr) ν: 3426 (NH), 3077 (arom-CH), 2893 (aliph-CH), 1652 (CO), 1555 (olefinic group), 1154 (C-N).; Anal. Calcd for C_33_H_37_N_3_O_2_ (507.67): C, 78.07%; H, 7.35%; N, 8.28% Found: C, 77.91%; H, 7.16%; N, 8.16%.

### Measurement of the spectral behaviors of the investigated chemosensors

As solvents for the titration studies, deionized water and a spectroscopic grade of ethanol were employed. In ethanol, 1 × 10^−3^ M stock solutions of the chemosensors and metal chloride salts of Al^3+^, Cr^3+^, Mn^2+^, Fe^3+^, Co^2+^, Ni^2+^, Cu^2+^, Mg^2+^, Hg^2+^ and Zn^2+^ were prepared. The absorption and fluorescence spectra were acquired from the chemosensor's solution 8 × 10^−5^ M for **1** and 6 × 10^−5^ M for **2** in a 1 cm quartz optical cell at ambient temperature. The trials were carried out after 10 min of mixing to guarantee homogeneity and balance. The concentration of the chemosensors was held constant during the titration while the concentration of metal ions was changed.

### Antioxidant activity

#### DPPH free radical scavenging activity

A modified Zheleva-Dimitrova technique was used to examine the DPPH free radical scavenging capabilities of the synthesised chemosensors^20^. Concisely, 25 µL from the successive concentrations of the synthesized chalcones (1.56–50 µg/mL) were mixed with 975 µL of (0.003 g %) DPPH^**∙**^ solution and the absorbance was measured at a wavelength of 515 nm after 1 h in dark room temperature incubation. l-ascorbic acid (AA; 2–20 µg/mL) was employed as a standard positive control and the DPPH^**∙**^ radical scavenging activity (%) was computed using Eq. (1):

1 $$\mathrm{DPPH\, radical \,scavenging \,activity}\mathbf{\%}=\frac{\mathrm{A\, control}-\mathrm{A \,sample}}{\mathrm{A\, control}} \times 100.$$

The compounds’ IC_50_ values were performed using GraphPad Prism 6 software.

#### ABTS^+^ free radical scavenging activity

The 2, 2 azino-bis3-ethylbenthiazoline-6-sulfonic acid radical (ABTS^+^) was used to test the free radical scavenging activity using a modified Re R. method^21^. Briefly, by reacting 14 mM ABTS^+^ solution with 4.9 mM potassium persulfate solution for 16 h in the dark, ABTS^+^ cation radicals were produced. The ABTS^+^ solution was diluted with distilled water to achieve an absorbance of 0.734 at 734 nm prior to use. Consequently, 25 µL from the serial concentrations of these synthesized chalcones (1.56–50 µg/mL) were added to 975 µL of ABTS^+^ solution. The mixture was vigorously shaken before being set aside for 6 min at room temperature, then the decrease in absorbance of the resultant solution was spectrophotometrically measured at 734 nm. L-ascorbic acid (AA; 2–20 µg/mL) was used as a positive control and the ABTS^+^ cation radical scavenging activity (%) was calculated using the Eq. (2):

2 $$\mathrm{ABTS \,radical\, scavenging \,activity}\left(\mathbf{\%}\right)=\frac{{\mathrm{A}}_{\mathrm{control}}-{\mathrm{A}}_{\mathrm{sample}}}{{\mathrm{A}}_{\mathrm{control}}}\times 100.$$

The compounds’ IC_50_ values were performed using GraphPad Prism 6 software.

### In-silico and ADMET studies

Molecular docking experiments were performed in this work to evaluate the binding mechanisms of chemosensor molecules to the target protein Akt. The targets’ crystal structures were obtained from the RCSB protein data (PDB:4EJN) (https://www.rcsb.org/structure/4EJN). To improve the target protein's efficiency, co-crystallized ligands, heteroatoms, and water molecules were eliminated. Furthermore, the produced analogues' 2D structures were constructed in cdx format (2D structures) and then converted to motif files (3D structures) using ChemDraw Ultra 8.0 (https://en.freedownloadmanager.org/users-choice/Chemdraw_Ultra_8.0. html). The enzyme-ligand interaction was investigated using Molegro Virtual Docker (2008) (http://molexus.io/molegro-virtual-docker/). Using the Discovery Studio 3.5 program (https://discover.3ds.com/discovery-studio-visualizer-download), the intermolecular interactions between the Akt protein and synthesized chemosensors were perceived^22,23^. The SwissADME online tool (http://www.swissadme.ch/) was also used to estimate absorption, distribution, metabolism, excretion, and toxicity (ADMET) factors that are important for drug design^24,25^.

### Anticancer assessments (in-vitro)

The MTT test was used to investigate the anticancer impact of the synthesized chemosensors in docking stimulations.

#### Cell lines maintenance, and treatment

The triple-negative breast cancer cell line MDA-231 (#ATCC HTB-26), colon cancer cell line (Caco-2) (#ATCC HTB-37), pancreatic cancer cell line (PANC-1) (#ATCC CRL-1469), and estrogen receptor-positive breast cancer cell line (MCF-7) (#ATCC HTB-22) cancer cell lines and WISH normal cells (#ATCC CCL-25) were separately seeded in 96 well plates with a complete media of (Dulbecco’s modified Eagle’s with 10% fetal bovine serum and 1% penicillin/streptomycin under a 5% CO_2_ and 95% humidified atmosphere at 37°C in a CO_2_ incubator). All cells were donated by Alexandria University's Centre of Excellence for Research in Regenerative Medicine and Its Applications. The cells were incubated for 48 h with chemosensors and Doxorubicin (DOX) as a reference drug at various concentrations (0–100 µM) then the viability of cells was measured using the tetrazolium 3-(4,5-dimethylthiazol-2-yl)-2,5-diphenyl-tetrazolium bromide (MTT) assay^26^.

### Statistical analysis

The data were expressed as the mean ± SE using GraphPad Prism software 6 (San Diego, CA) (GraphPad Prism 6, https://www.graphpad.com/scientific-software/prism/).

## Results and discussion

### Synthesis and characterization of the chemosensors

The starting compound 3,5-diacetyl-2,6-dimethyl-1,4-dihydro pyridine was generated with an 87% yield in a one-pot, three-component synthesis involving formaldehyde, acetyl acetone, and ammonium acetate. This molecule was subsequently treated with 4-(dimethylamino)benzaldehyde or 4-(dimethylamino)cinnamaldehyde to form chemosensors **1** and **2** with chemical yields of 87% and 88%, respectively, as shown in Fig. 1.

**Figure 2:**
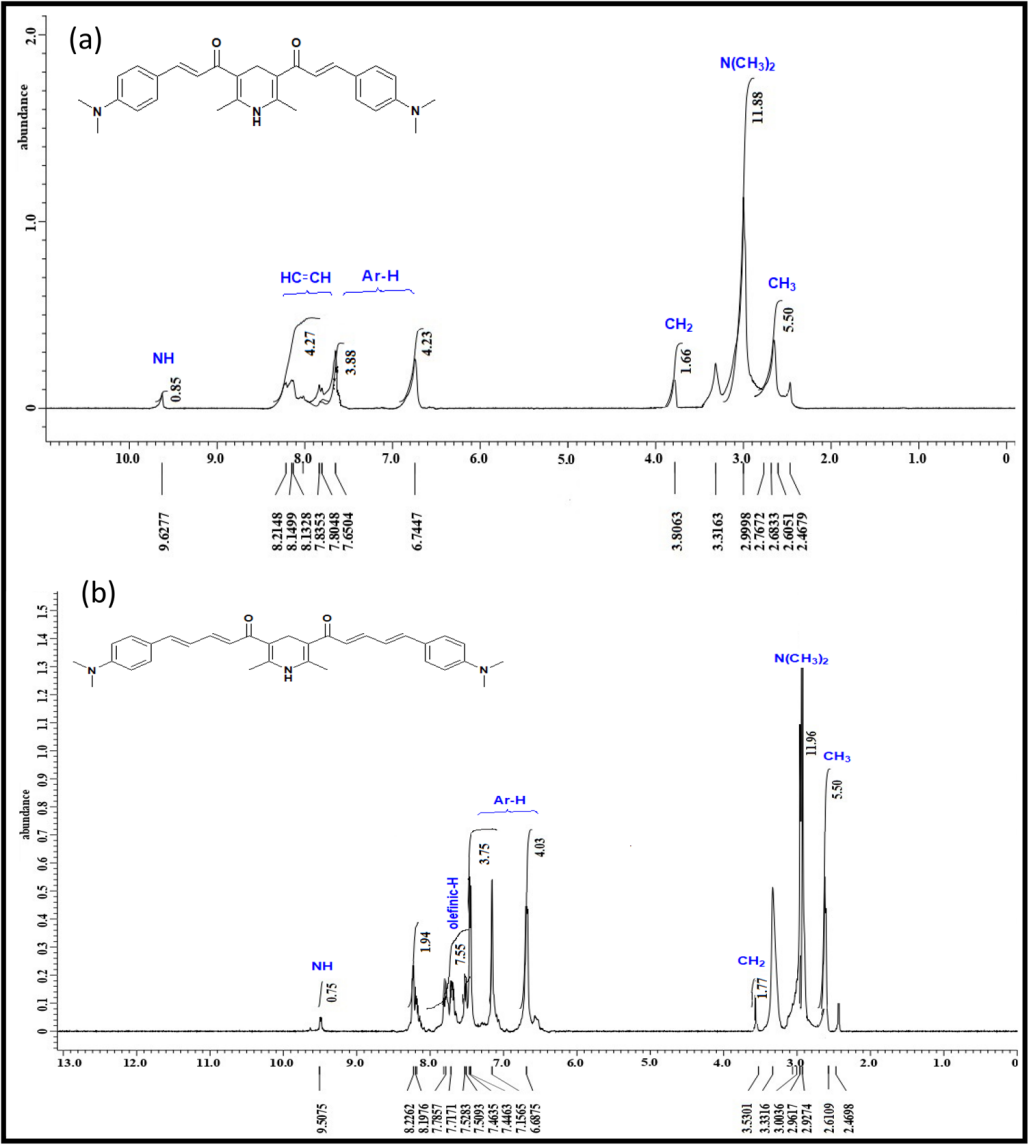
^1^H NMR spectra of chemosensors **1** and** 2**.

**Figure 3:**
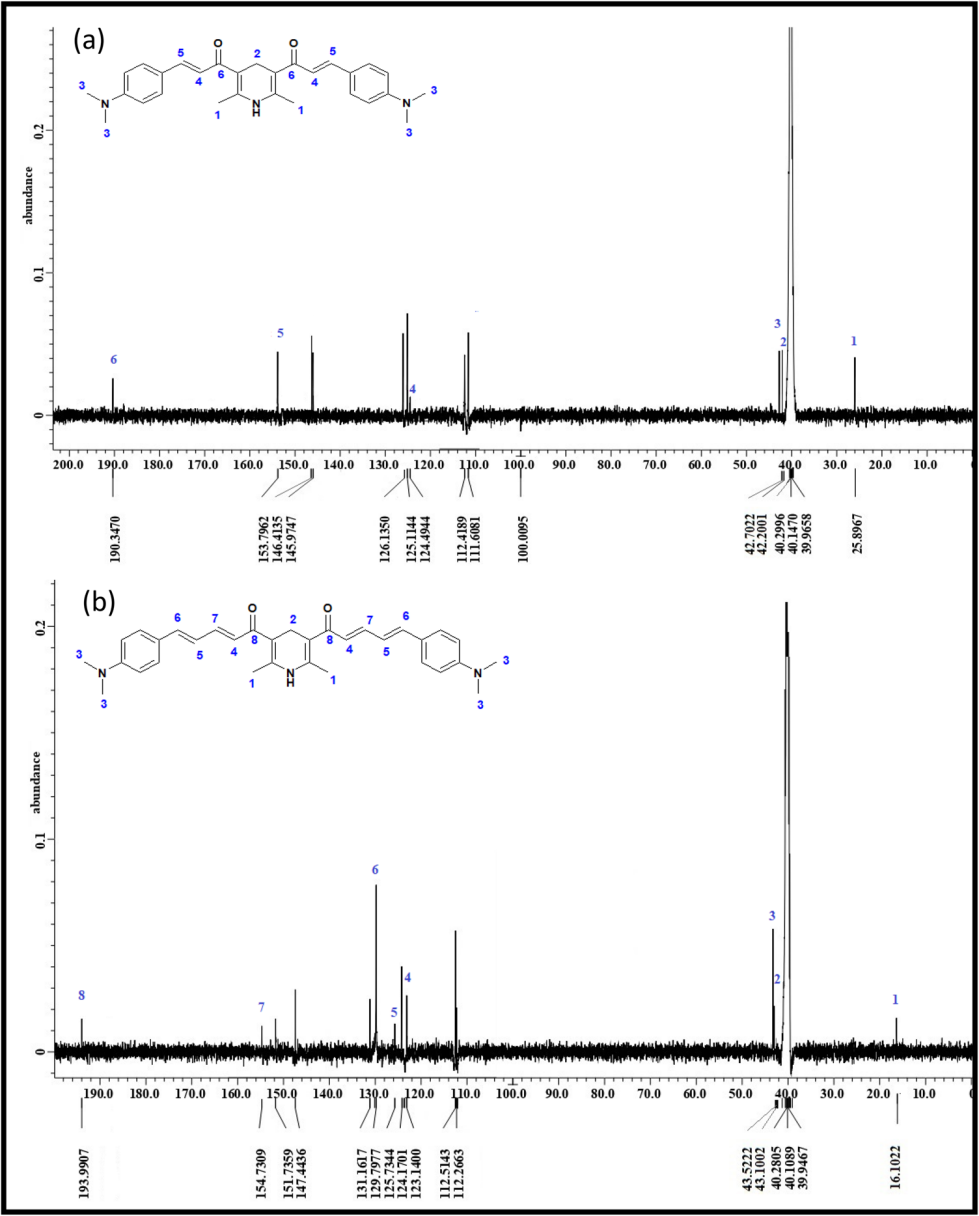
^13^C NMR spectra of chemosensors **1** and** 2**.

**Figure 4:**
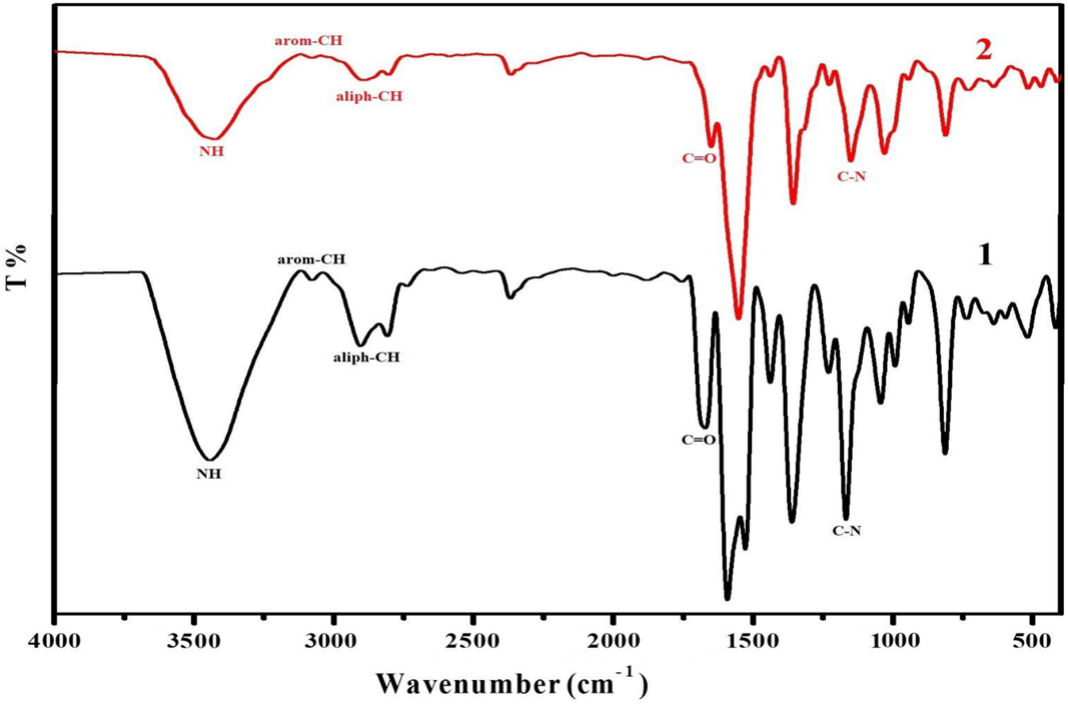
FT-IR spectra of chemosensors **1** and** 2**.

After flash column chromatography, the structures of synthesized chemosensors were characterized using ^1^H NMR (Fig. 2), ^13^C NMR spectra (Fig. 3), FT-IR spectra (Fig. 4) and elemental analyses.

### Solvatochromic behaviors of the investigated chemosensors in different solvents

**Figure 5:**
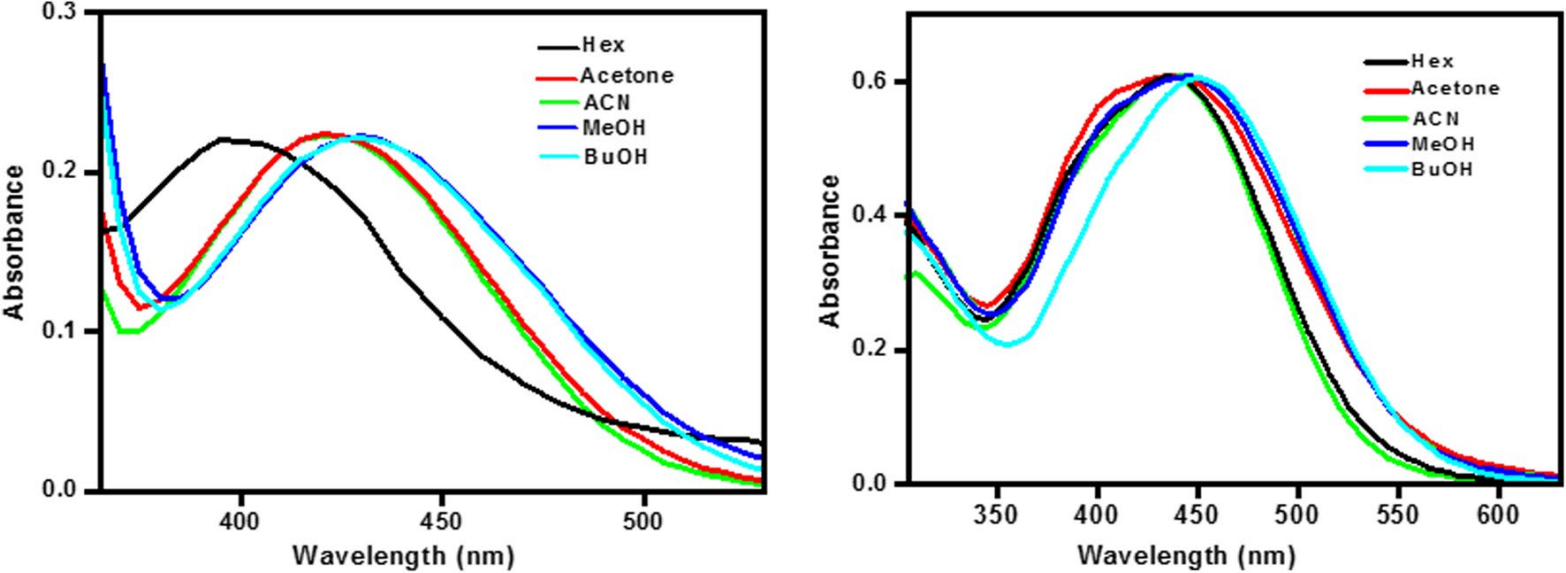
Absorption spectra of the investigated chemosensors in different solvents.

Figure 5 displayed the normalized absorption and fluorescence spectra of the tested chemosensors **1** and **2** at room temperature in solvents ranging in polarities such as: MeOH, EtOH, Iso PrOH, BuOH, ACN, DMF, CH_2_Cl_2_, CHCl_3_, acetone, Benz, toluene and Hex. The spectra broadened (expressed as band width at half maximum, **Δυ**_**1/2**_), as the polarity of the solvent increased. The strong long-wavelength absorption band of chemosensors **1** and **2** undergoes substantial red-shifts with increasing solvent polarity (*ca.* 96 and 22 nm on going from toluene to MeOH, respectively). These characteristics imply a highly permitted π–π* transition with charge transfer characters. Based on the chemical structure, the spectrum alterations might be attributed to intramolecular charge transfer (ICT) from the dimethylaniline moiety to the carbonyl oxygen in the ground state.

**Figure 6:**
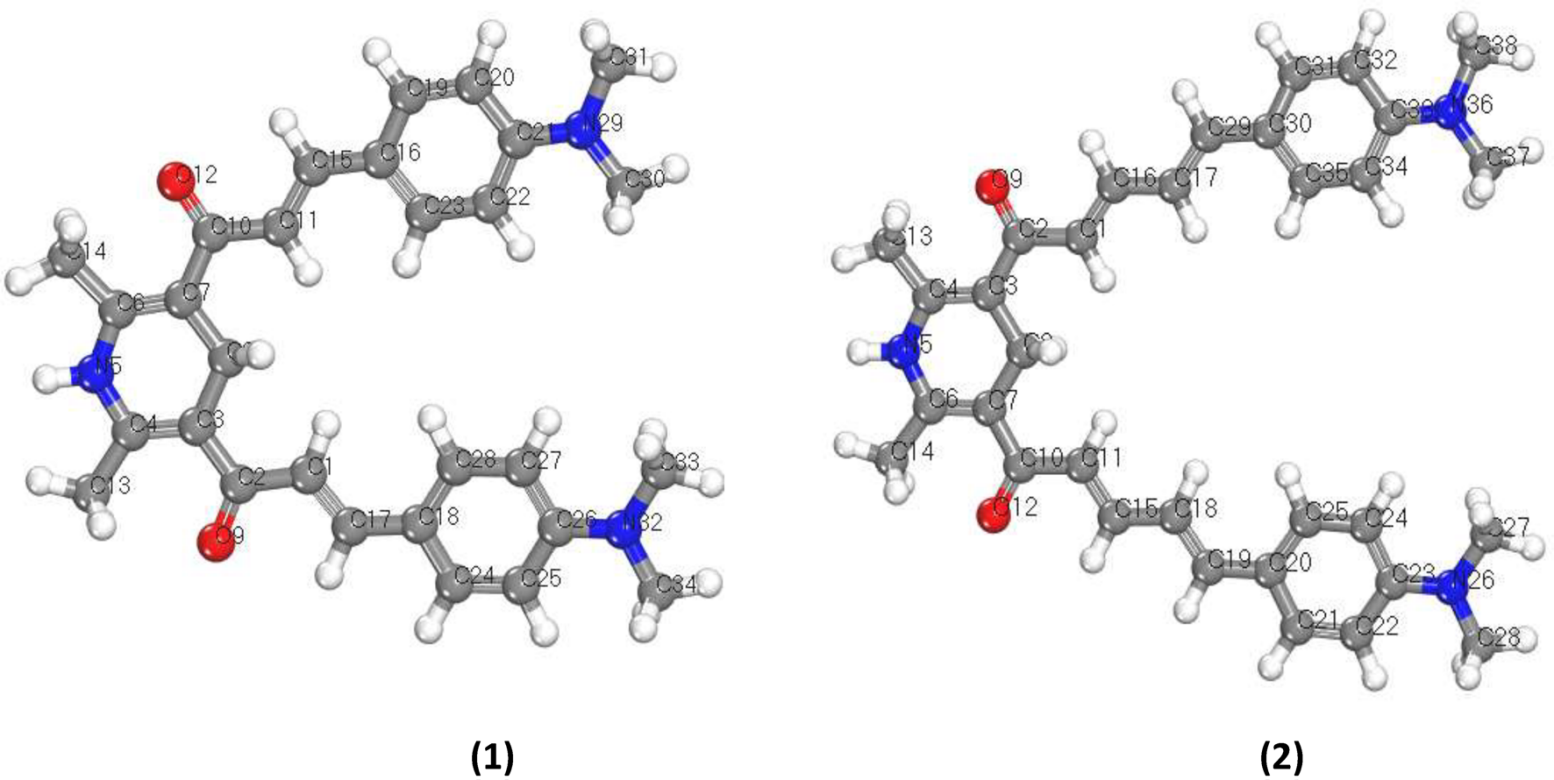
The optimized geometries of the chemosensors.

Theoretical computations were addressed in order to fully appreciate the spectrum alterations of the chemosensors. The density functional theory (DFT) computations were carried out using the DMol^3^ module in BIOVIA Inc.'s Materials Studio program (version 20.1)^27,28^. The atomic basis of the DMol^3^ program is numerical functions on an atom-centered grid, which is significantly more comprehensive than typical Gaussian functions. The hybrid functional of Becke-3-exchange plus Lee–Yang–Parr correlation (B3LYP) in conjunction with the double numerical with polarization (DNP) basis set were used to perform geometrical optimizations. Figure 6 showed the optimized geometries of the chemosensors **1** and **2**.

**Figure 7:**
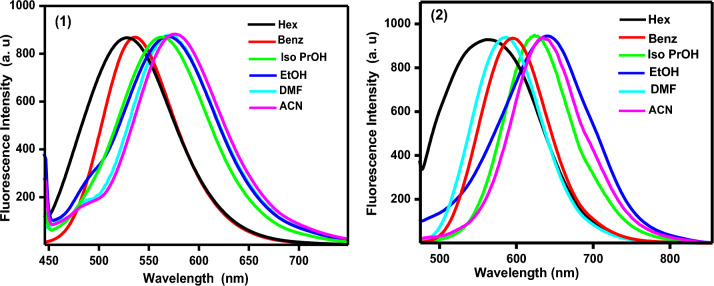
Emission spectra of the investigated chemosensors in different solvents.

Figure 7 depicted the normalized emission spectra of the chemosensors **1** and** 2** in various solvents, and Table 1 has the related fluorescence data. As can be seen, the fluorescence maxima of the chemosensors **1** and **2** exhibited a significant bathochromic shift as the solvent polarity increased from Hex to BuOH by 38 and 66 nm, respectively, pointing to the role of the contribution of photoinduced intramolecular charge transfer within the molecule in the singlet excited state. Furthermore, the rising Stokes shift as solvent polarity increases shows that the examined chemosensors fluoresce from relaxed to ground states, and the solvent relaxation duration is less than the radiative lifetimes. The amplitude of the Stokes shifts of the examined chemosensors varied greatly depending on the chemosensor construction and solvent polarity. The observed substantial Stokes shift values corroborate the charge transfer character of the long-wavelength transition and suggest that the excited state dipole moment is greater than the ground state dipole moment.

**Table 1:** Spectroscopic data of the chemosensor** 1** and **2** in various solvents of different polarities.

Solvent	Chemosensor 1					Chemosensor 2				
	$\lambda_{\max }^{{\text{a}}}$ nm	$\lambda_{\max }^{{\text{f}}}$ nm	$\Delta \overline{\nu }$ cm^–1^	$\varepsilon \times 10^{4}$ L mol^–1^ cm^–1^	$\Phi_{f}$	$\lambda_{\max }^{{\text{a}}}$ nm	$\lambda_{\max }^{{\text{f}}}$ nm	$\Delta \overline{\nu }$ cm^–1^	$\varepsilon \times 10^{4}$ L mol^–1^ cm^–1^	$\Phi_{f}$
Toluene	334	570	12,396	2.0	0.08	420	584	6686	5.0	0.30
Benz	420	536	5152	1.9	0.03	445	596	5693	5.0	0.05
Hex	397	529	6285	2.1	0.002	436	565	5236	6.1	0.01
Acetone	422	566	6028	2.1	0.017	439	537	4157	5.2	0.02
DMF	429	570	5766	3.0	0.02	447	624	6345	7.4	0.05
CH_2_Cl_2_	429	559	5420	1.5	0.10	447	621	6268	4.5	0.15
CHCl_3_	429	550	5128	2.5	0.20	447	610	5977	9.2	0.60
CAN	422	578	6395	2.2	0.006	435	536	4331	6.4	0.002
BuOH	430	567	1842	2.0	0.04	450	631	6374	6.4	0.074
IsoPrOH	428	563	5602	1.8	0.03	442	634	6851	6.0	0.07
EtOH	430	568	5650	2.0	0.012	445	640	6846	6.5	0.002
MeOH	430	572	5773	2.2	0.005	442	647	7168	5.2	0.001

Table 1 summarizes the fluorescence quantum yield (Φ_f_) of the examined chemosensors recorded in various solvents with varied polarity. The findings revealed that Ф_f_ values are highly reliant on the composition of the solvent. In chlorinated solvents such as CHCl_3_, the Ф_f_ values for chemosensors **1** and **2** are 0.2 and 0.6, respectively. Furthermore, the measured Ф_f_ values in non-polar solvents such as Hex are 0.002 and 0.01, respectively. This demonstrates the importance of molecule structure and chain length in causing major spectrum alterations. The solvent dependency of the fluorescence quantum yield was also investigated using a multi-solvent parameter method, which included the solvent acidity (SA), basisity (SB), polarizability (SP), and dipolarity (SdP)^29^. In the light of this analysis, the following equation has been obtained:

$${\mathbf{Chemosensor}} \, {\mathbf{1}}; \, \Phi_{{\text{f}}} = \, - 0.{353} + \, 0.{\text{111 SA}} - 0.{1}0{\text{5 SB }} + \, 0.{\text{58 SP }} + \, 0.0{\text{31SdP}},$$

$${\mathbf{Chemosensor}} \, {\mathbf{2}};\Phi_{{\text{f}}} = \, - 0.{96 } + \, 0.{\text{33 SA}} - 0.{\text{29 SB }} + { 1}.{\text{57 SPP}} + \, 0.0{\text{56SdP}}.$$

The individual contribution of distinct forms of solute–solvent interactions that influence the solvent-induced spectrum shifts was assessed using a multiparametric methodology known as the solvatochromic comparison method (SCM), which was introduced by Kamlet et al.^30^. This method distinguishes the dielectric effects of solvents (π*), hydrogen-bond donor (α) and acceptor (β) abilities of solvents on spectral characteristics. The SCM was used to analyse the absorption and emission maxima based on the following correlations:

**Table:** 

**For chemosensor 1**	${\overline{{\varvec{\nu}}} }^{{\varvec{a}}}$= 24889 – 862 α + 85 β – 1782 π* ${\overline{{\varvec{\nu}}} }^{{\varvec{f}}}$= 31240 + 19434 α + 65009 β–49152π*
**For chemosensor 2**	${\overline{{\varvec{\nu}}} }^{{\varvec{a}}}$= 23083–426 α – 133 β–366 π* ${\overline{{\varvec{\nu}}} }^{{\varvec{f}}}$= 17688–2087 α + 151 β–533π*

From the above equations, it could be observed that the absorption and emission were controlled by the antagonizing effects of the different parameters. Also, the chemosensor 1 was affected significantly by α, β, and π* coefficient compared to the chemosensors **1**, and the negative sign confirms the cooperative effects of all modes.

**Figure 8:**
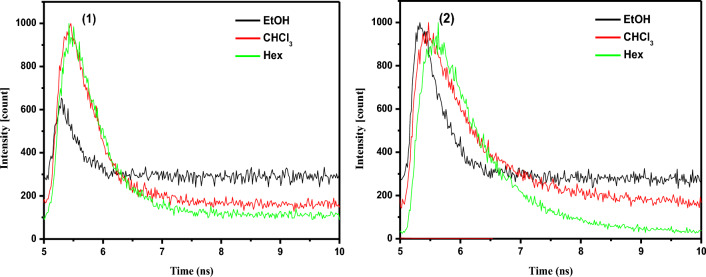
Normalized time resolved fluorescence decay for the investigated chemosensors in EtOH, CHCl_3_ and Hex.

We performed time-resolved fluorescence experiments in three distinct solvents, EtOH, CHCl3, and Hex, to elucidate the nature of the excited state of the examined chemosensors (Fig. 8), and the results were gathered in Table 1. In the employed solvents, the fluorescence decay profiles of chemosensors **1** and **2** were best suited to mono-exponential decay, except for chemosensor **2** in CHCl_3_, where the emission decay curves were well fitted with a double-exponential function. The fluorescence from the non-relaxed excited state is most likely associated with the short lifetime component, whereas the nanosecond fluorescence lifetime is associated with the relaxed state of CT character.

The rate constants of the radiative (*k*_r_) and nonradiative (*k*_nr_) processes of the excited dyes were computed from the Ф_f_ and lifetime values using *k*_r_ = ϕ_f_∕τ_f_ and *k*_nr_ = (1–ϕ_f_*)*/τ_f_^31^, and the assessed results were summarized in Table 2. As shown in Table 2, the radiative and nonradiative rate constants of the chemosensor **1** fall dramatically as the medium polarity decreases from EtOH to Hex. In the case of the chemosensor **2**, however, when the solvent polarity diminishes, both radiative and nonradiative constants rise.

**Table 2:** Fluorescence lifetimes, radiative and non-radiative constants of the investigated chemosensors in three different solvents EtOH, CHCl_3_ and hex.

Chemosensor	Solvent	Lifetime, τ (ns)	χ^2^, %	K_r_ × 10^9^ s^–1^	Kn_r_ × 10^9^ s^–1^
**1**	EtOH	0.016	100	0.75	61.75
	CHCl_3_	0.253	100	0.79	3.16
	Hex	0.311	100	0.006	3.21
**2**	EtOH	0.135	100	0.01	7.39
	CHCl_3_	0.882	70.39	0.68	0.453
		0.114	29.61	5.2	3.51
	Hex	0.001	100	9.0	991.0

To analyze the influence of molecular structure on the spectrum behavior of the researched chemosensors, the absorption and fluorescence spectra of these chemosensors will be addressed in EtOH as an example. The acquired results revealed that depending on the structure of the utilized chemosensors, there is a considerable bathochromic change in the absorption maximum by lengthening the area of electron delocalization. Increased conjugation lengthens the region of electron delocalization, resulting in a reduction in the energy gap between the ground and excited states. Accordingly, a progressive shift in both absorption and emission maxima is seen, where the substitution of dimethyl 4-vinyl benzenamine fragment in chemosensor **1** by 4-but-1,3-dienyl-diamethyl benzenamine fragment in chemosensor **2** boosted the resonance delocalization of the π -electrons. The red shift in the absorption and the emission spectra was (430, 445 nm) and (568, 640 nm) for **1** and **2**, respectively. Also, the fluorescence quantum yield is strongly dependent on the molecular structure.

### Colorimetric assay of the investigated chemosensors towards the metal ions

**Figure 9:**
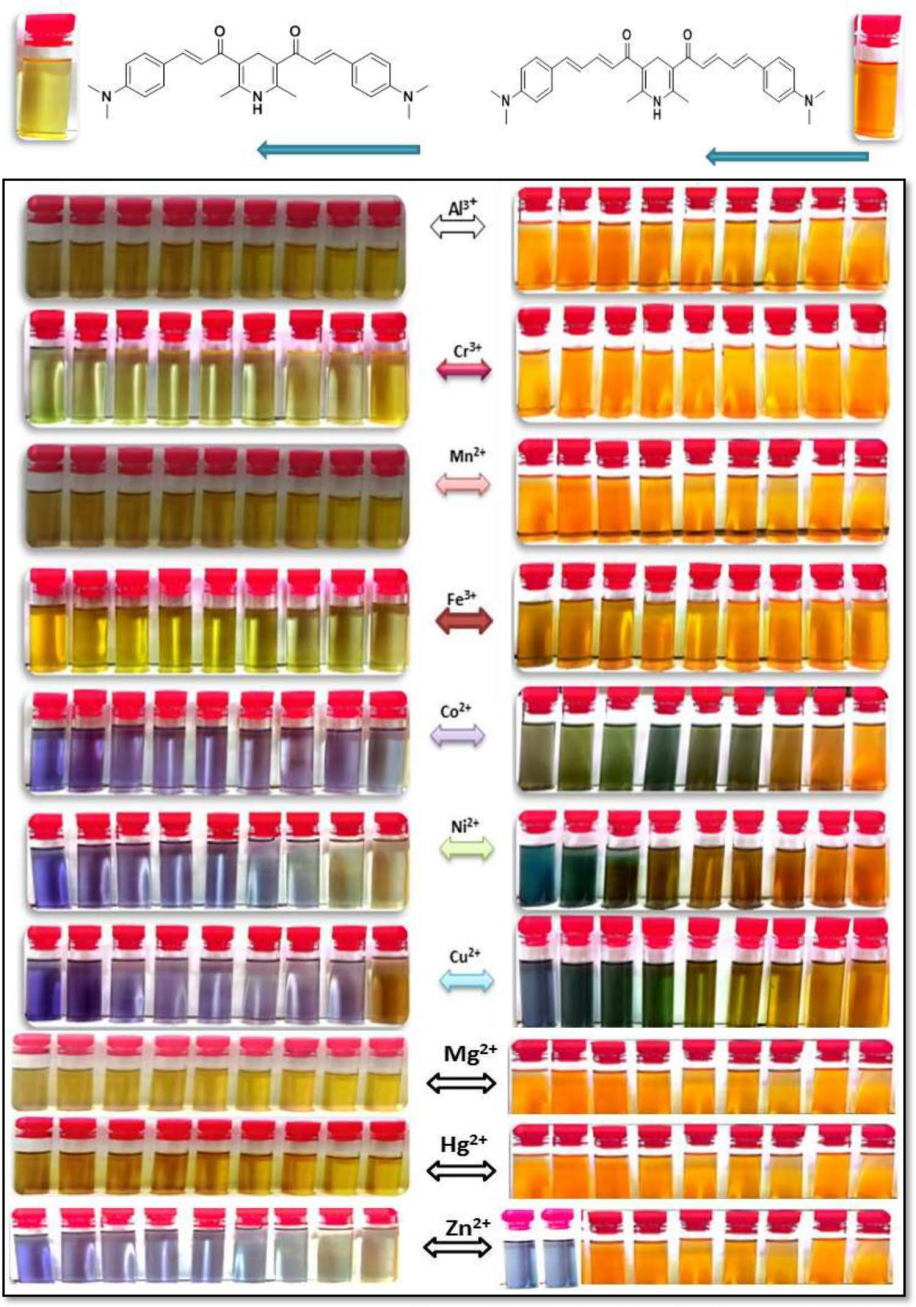
Color changes in the absence and presence of various concentrations of metal ions (0–5.4 × 10^–5^ M) to the chemosensors **1** and **2**.

The cost effectiveness, colorimetric and detection capabilities of the chemosensors **1** and **2** towards different various metal ions such as Al^3+^, Cr^3+^, Mn^2+^, Fe^3+^, Co^2+^, Ni^2+^, Cu^2+^, Mg^2+^, Hg^2+^ and Zn^2+^ were investigated in ethanolic solution at room temperature. As shown in Fig. 9, the chemosensors showed an observable change in the color in the absence and presence of varied concentrations of the specified metal ions (0–5.4 × 10^–5^ M), indicating that the chemosensors can serve as a potential candidate of naked-eye detection in ethanolic solution.

**Figure 10:**
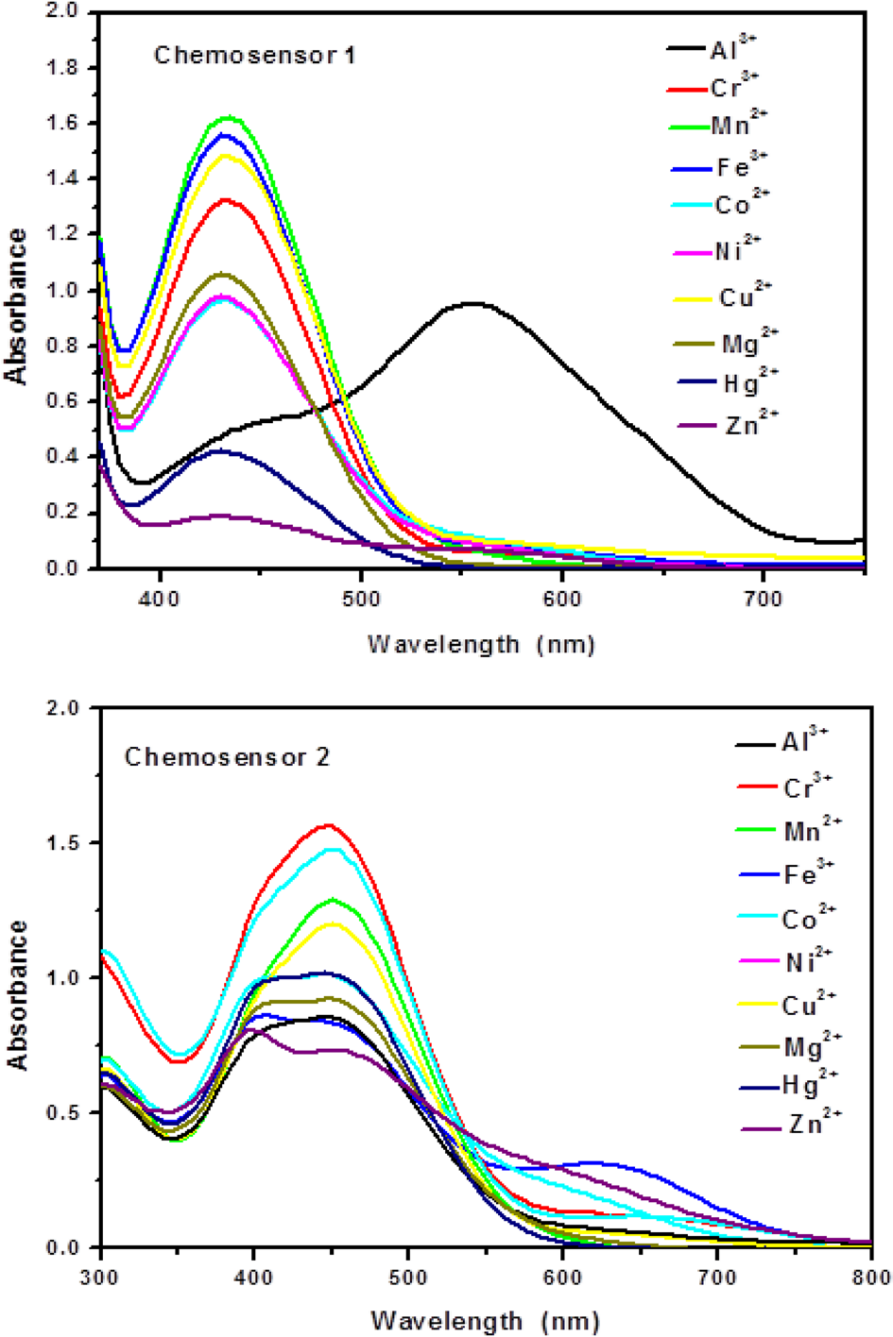
Changes in the absorbance of the chemosensors (2 × 10^–5^ M) upon addition of a particular metal salt (6 × 10^–5^ M) in ethanolic solution.

**Figure 11:**
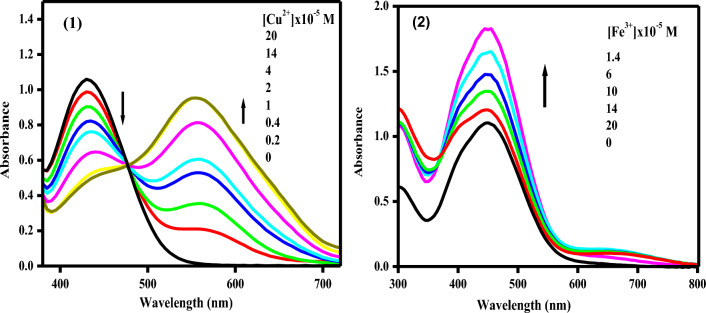
Absorption spectral changes of the chemosensors **1** and **2** in the presence of different concentrations of Cu^2+^ and Fe^3+^ ions, respectively in ethanolic solution.

In ethanolic solution, the absorption spectra of free chemosensors **1** and **2** showed high absorption bands at 430 and 445nm (ε = 2.65 and 2.70 × 10^4^ L mol^–1^cm^–1^, respectively). As can be shown in Fig. 10, the examined chemosensors **1** and **2** demonstrated selectivity to Cu^2+^ and Fe^3+^ ions, respectively, when compared to other metal ions. The chemosensors' selectivity for metals might be owing to differences in the size and charge density of the metal ions. In Fig. 11, the absorption spectrum significantly changed when different concentrations of Cu^2+^ ions were added to chemosensor **1**. A hypochromic effect was observed at 430 nm and a hyperchromic effect was observed at 555 nm, with the formation of an isosbestic point at 476 nm, suggesting the complex formed upon binding of chemosensor **1** to Cu^2+^ ions. Also, the chemosensor **2**'s absorption band at 445 nm steadily grew and a new visible band at 653 nm was generated after the addition of Fe^3+^ ions. This could be due to the coordination between the mentioned metal ions and the carbonyl groups of the chemosensors causing ligand-to-metal charge transfer (LMCT).

**Figure 12:**
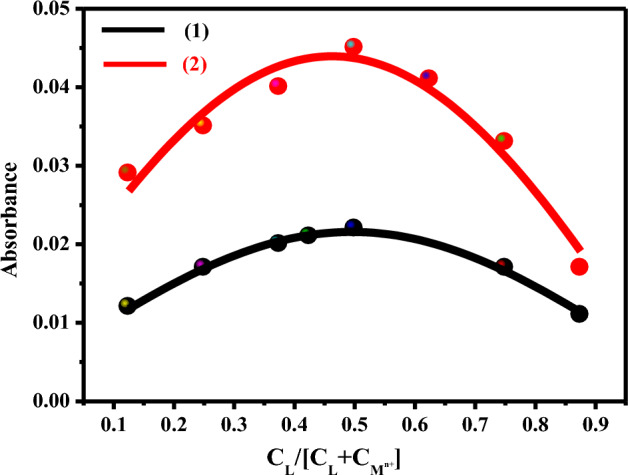
Job’s plot for the investigated chemosensors **1** and **2** with Cu^2+^ and Fe^3+^ ions, respectively.

Job’s plot analysis revealed a 1:1 stoichiometry for the binding of chemosensors **1** and **2** to the ions Cu^2+^ and Fe^3+^, respectively (Fig. 12). The Benesi-Hildebrand equation was used to compute the binding constant of the complexes that were generated between the metal ions and the chemosensors^32^. Table 3 lists the binding constants after graphing 1/(A − A_o_) versus 1/ [M^n+^] Fig. S5. The results collected revealed that, compared to the other metals, the chemosensors **1** and **2** had the greatest binding constants for the ions Cu^2+^ (13,222 M^–1^) and Fe^3+^ions (82,523 M^–1^), respectively.

**Table 3:** Spectral data, binding constants in both ground and excited states, Stern–Volmer constants and limit of detection (LOD) for analytical method validation using **1** and **2** as colorimetric sensors.

Metal ions	Chemosensor 1						Chemosensor 2					
	$\lambda_{\max }^{{\text{a}}}$ nm	$\lambda_{\max }^{{\text{f}}}$ nm	${\text{K}}_{{\text{b}}}^{{\text{a}}}$(M^–1^)	${\text{K}}_{{{\text{SV}}}}$(M^–1^)	LOD × 10^–4^ (M)	K_q_ × 10^13^ (M^–1^S^–1^)	$\lambda_{\max }^{{\text{a}}}$ nm	$\lambda_{\max }^{{\text{f}}}$ nm	${\text{K}}_{{\text{b}}}^{{\text{a}}}$(M^–1^)	${\text{K}}_{{{\text{SV}}}}$(M^–1^)	LOD × 10^–4^ (M)	K_q_ × 10^13^ (M^–1^S^–1^)
Free	430	571	–	–	–	–	445	647	–	–	–	–
Al^3+^	435	573	3950	1932.5	0.022	12.10	452	641	27,308	–	1.21	–
Cr^3+^	601	571	1283	52.8	0.22	0.33	620	631	171	141.4	6.17	0.105
Mn^2+^	436	569	5657	–	0.49	–	452	648	4498	1600.7	4.51	1.18
Fe^3+^	591	565	6770	942.8	4.69	5.89	653	605	82,523	1090.8	6.73	0.808
Co^2+^	570	576	710	255.28	4.70	1.59	613	654	278	–	4.10	–
Ni^2+^	577	559	1439	624.9	0.14	3.90	626	645	4884	328.3	4.46	0.243
Cu^2+^	555	579	13,222	2997.0	1.64	18.7	608	644	60,784	1914.9	3.62	1.418
Mg^2+^	429	569	1387	706.01	1.5	4.41	445	641	–	–	–	–
Hg^2+^	431	570	245	549.5	0.48	3.43	445	640	685	298.0	2.13	0.22
Zn^2+^	561	565	812	624.4	3.38	3.90	445	642	528	402.3	0.96	0.298

**Figure 13:**
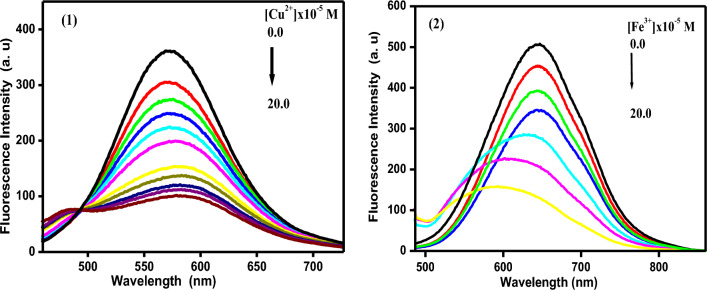
Fluorescence spectra of the chemosensors in the absence and presence of different concentrations of some selected metal ions in EtOH.

The examined chemosensors **1** and **2**’s fluorescence spectra were displayed in Fig. 13 after the addition of various concentrations of Cu^2+^ and Fe^3+^ions within the ranges (0–2 × 10^–5^ M, respectively). The emission bands at 571 and 647 nm in EtOH were seen on the chemosensors **1** and **2**. The fluorescence intensity slowly decreases with a bathochromic shift of 9 nm as varied amounts of Cu^2+^ ions are added to the chemosensor **1**. However, the fluorescence intensity is quenched with a discernible hyposchromic shift of 42 nm when Fe^3+^ ions are added to the chemosensors **2**.

The Stern–Volmer constants were computed using the following equation from the fluorescence quenching data^33^:

$${\text{F}}_{{\text{o}}} /{\text{ F}}_{{\text{x}}} = { 1} + {\text{ K}}_{{{\text{sv}}}} \left[ {{\text{M}}^{{{\text{n}} + }} } \right] \, = {\text{k}}_{{\text{q}}} {\text{t}}\left[ {{\text{M}}^{{{\text{n}} + }} } \right],$$

where, F_o_ and F_x_ are the fluorescence intensities both when the investigated metal ions are absent and present (M^n+^), K_SV_ is the Stern–Volmer constant and k_q_ is the quenching rate constant, τ is the lifetime of the chemosensors in the absence of the quenchers, metal ions. In Fig. S6, the Stern–Volmer plots for chemosensors **1** and **2** were presented. The plots unmistakably displayed increased curvatures with greater metal ion concentrations. These curvatures support the notion that the quenching process results from the creation of a ground state complex between the examined chemosensors and the analyzed metal ions. The values of K_SV_ and k_q_ are listed in Table 3. The values of quenching constant are ranging from 0.09 to18.7 × 10^13^ M^−1^s^−1^^34^. For dynamic quenching, the maximum value of quenching constants is 2.0 × 10^10^ M^−1^s^−1^^35^. Additionally, the stronger quenching of the chemosensors **1** and **2** by Cu^2+^ and Fe^3+^ ions, respectively, over the other metal ions is shown by the larger K_SV_ values.

Based on the results of the fluorescence titration profile, detection limits were also computed and summarized in Table 3. The detection limits for Cu^2+^ and Fe^3+^ using chemosensors **1** and **2**, respectively, exceeded the World Health Organization's (WHO) drinking water guidelines^36^, but were relatively low when compared to the values published for other organic chemosensors^37–40^. The Stern–Volmer constants show that the chemosensors **1** and **2** can function as practical tools for detecting the indicated metal ions without costly apparatus.

**Figure 14:**
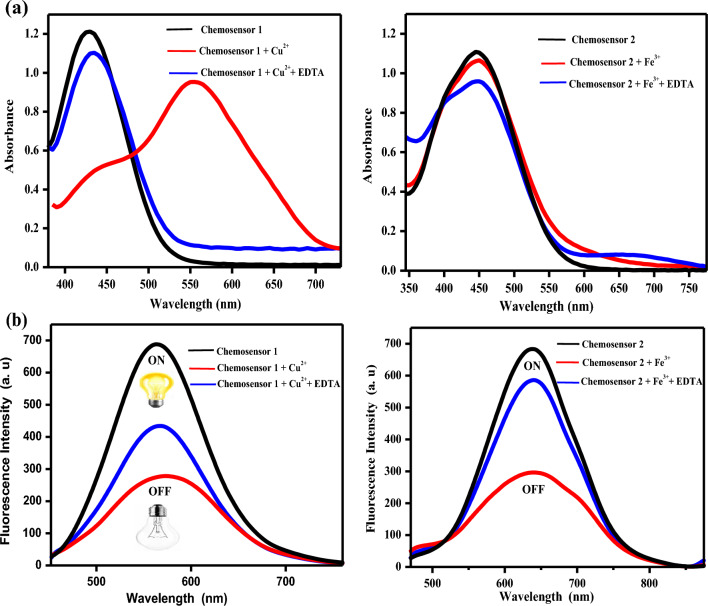
(**a**) Absorption and (**b**) Fluorescence spectra of the free chemosensors and their complexes in absence and presence of EDTA.

**Figure 15:**
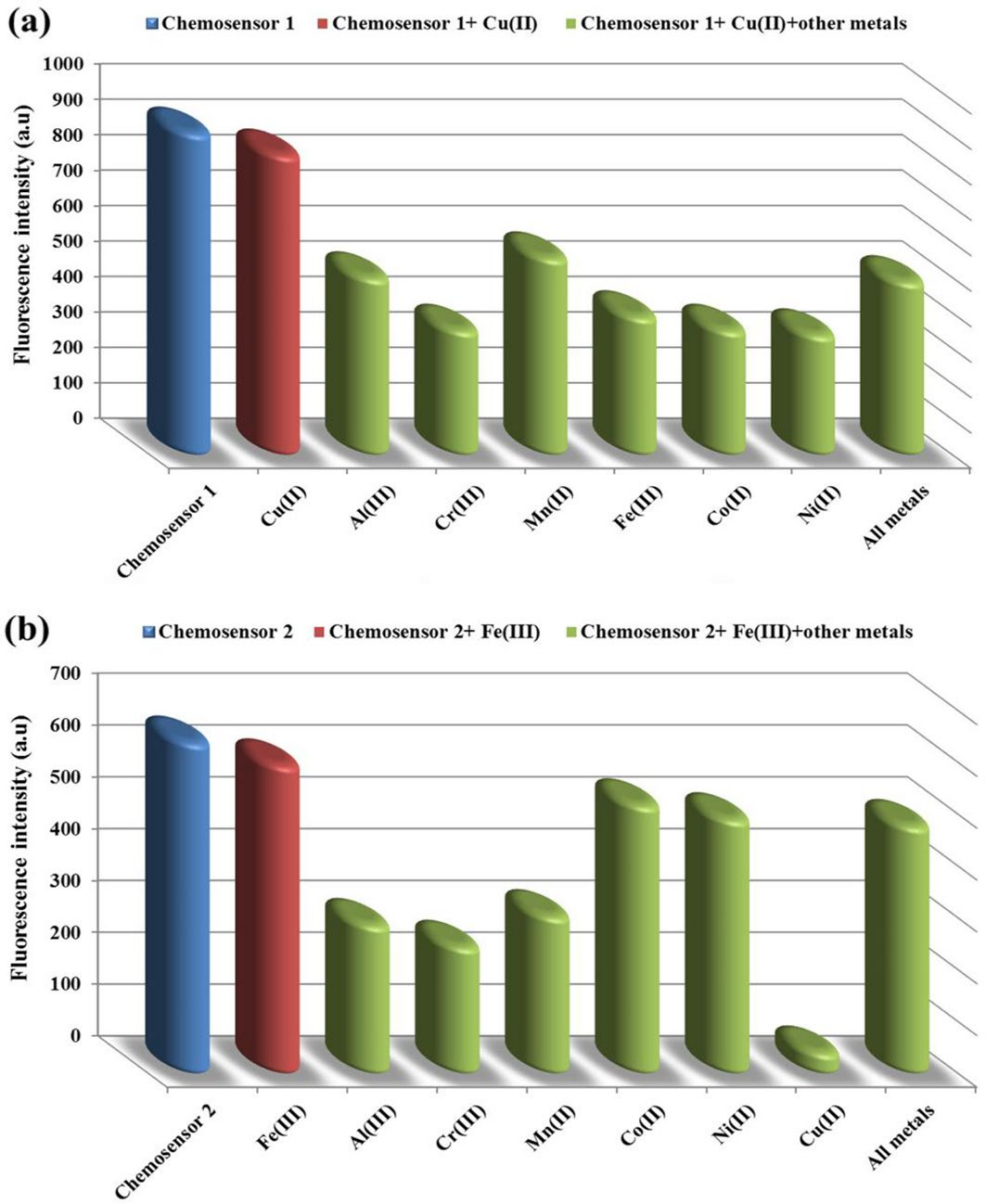
(**a**) Selectivity of chemosensor **1** to Cu^2+^ ions and (**b**) chemosensor **2** to Fe^3+^ ions with the different metal ions in ethanolic solution at the same concentration (6 × 10^−4^ M).

Ethylenediaminetetraacetic acid (EDTA, 1 × 10^–5^ M) was introduced to a mixture of the chemosensors and the afore mentioned metal ions in order to test the reversibility of the chemosensors **1** and** 2** toward Cu^2+^ and Fe^3+^ ions, respectively. The addition of EDTA to the generated complex led to the restoration of the color of the original solutions of the free chemosensors, as shown in Fig. 14a,b, demonstrating the complete reversibility of the optical response to the examined metal ions. The original absorption and emission spectra were restored by the metal ion chelator, and the chemosensors are generally in a switched-on state. Adding metal ions to the solution allowed for the recovery of the color, absorbance, and emission. These findings suggest that the chemosensors might be employed in ethanolic solution as a colorimetric reversible on–off-on chemosensor. In the presence of the other metal ions, the preferred selectivity of the chemosensors **1** and **2** as colorimetric sensors for Cu^2+^ and Fe^3+^ ions, respectively, was studied. The detection of the afore mentioned metal ions was slightly hampered when the chemosensors **1** and **2** were exposed to 5.5 × 10^−6^ M of Cu^2+^ and Fe^3+^ ions, respectively, in the presence of the same concentration of the interfering metal ions, as shown in Fig. 15a,b. This finding suggested that the potent chemosensors** 1** and **2** may be utilized to detect Cu^2+^ and Fe^3+^ ions in ethanolic solution.

### Antioxidant capacity

**Figure 16:**
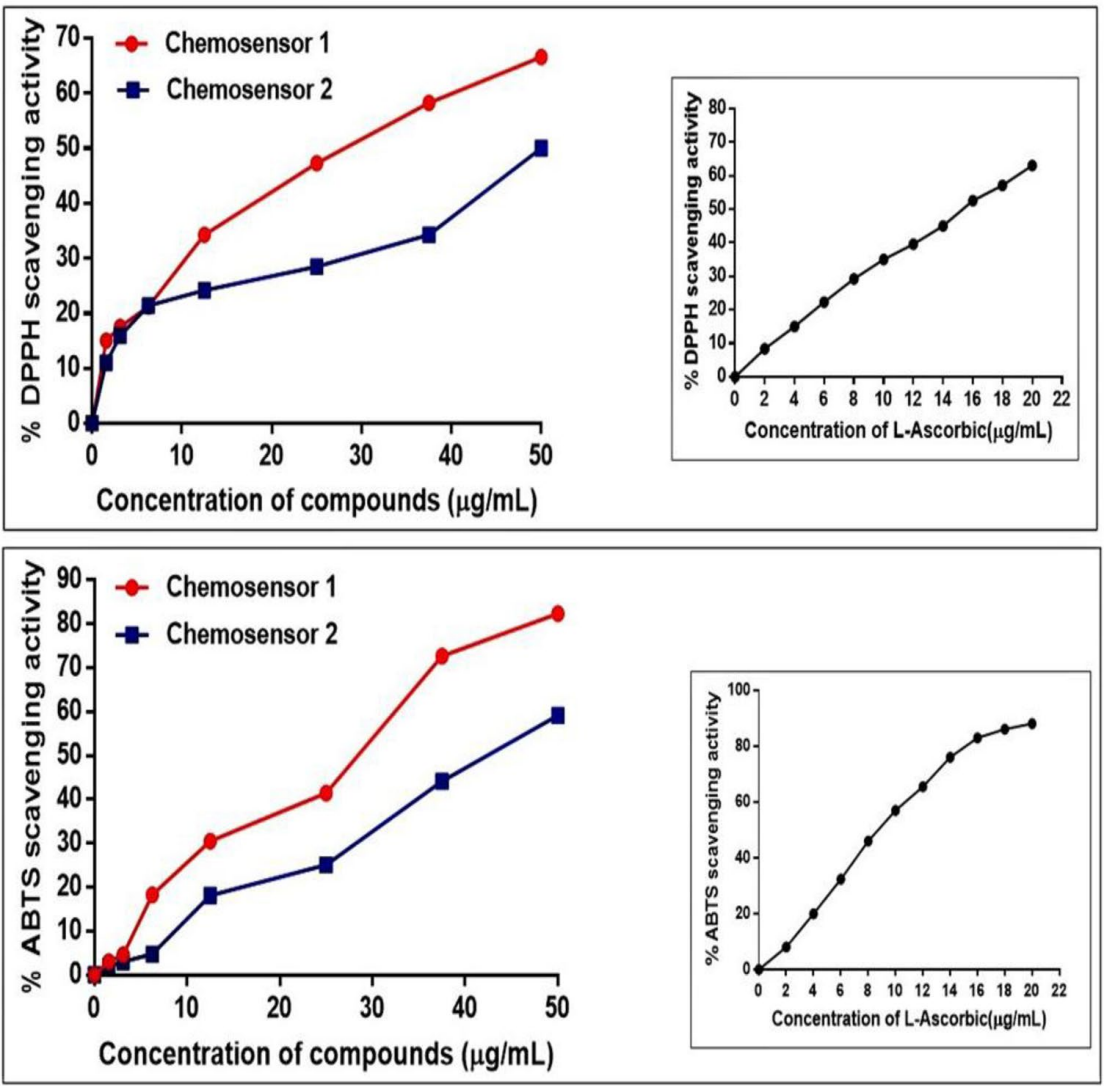
The DPPH^**∙**^ and ABTS^+^ antioxidant scavenging activity of chemosensors **1** and** 2** compared with the standard l-ascorbic acid.

**Figure 17:**
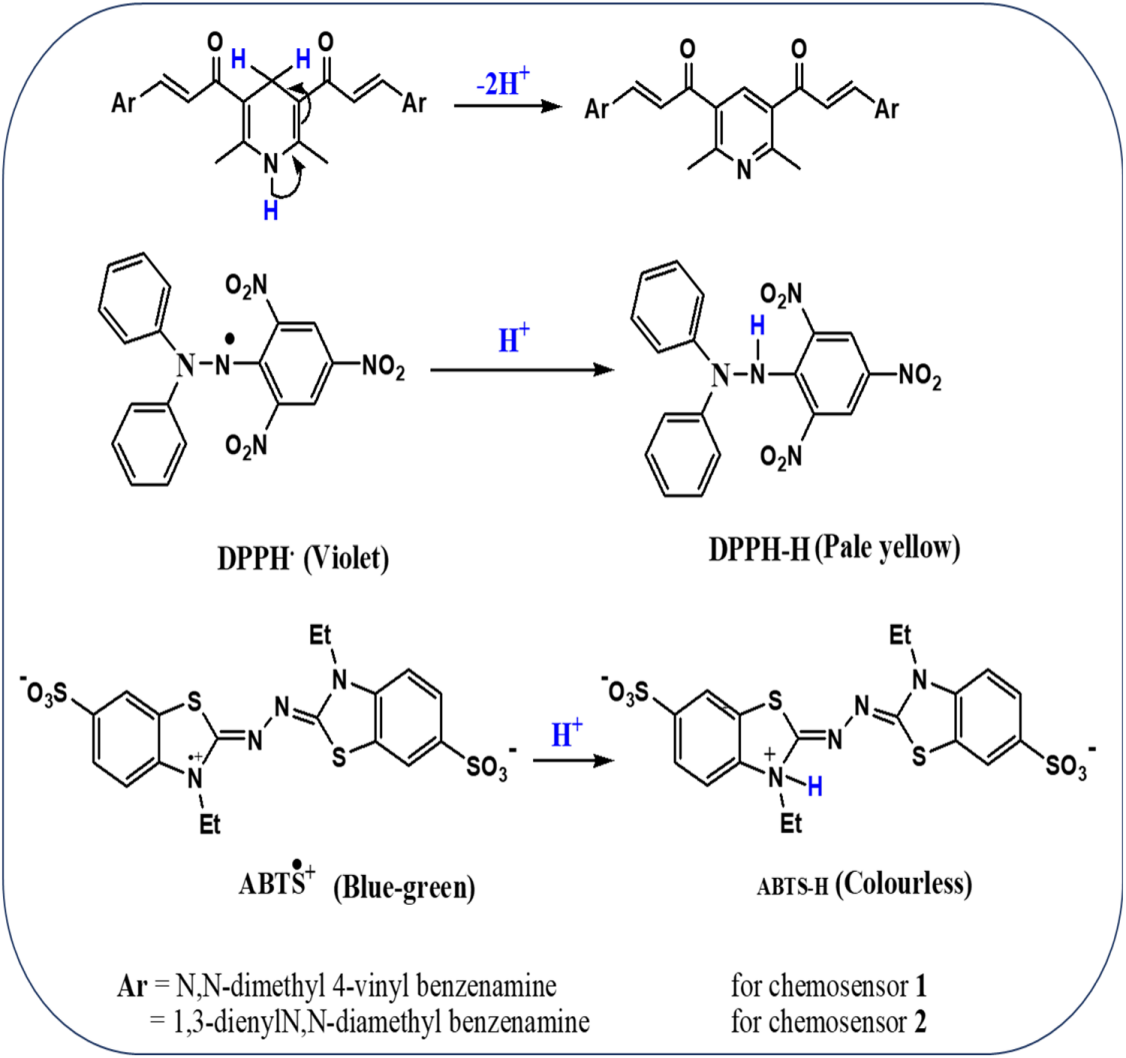
Mechanism of redox reaction between the chemosensors and the investigated radicals.

The antioxidant capability of these recently synthesised chalcones was evaluated using the stable DPPH^**∙**^ and ABTS^+^ radical scavenging activity tests by observing the change in absorbance generated, as shown in Fig. 16. The data obtained indicated that as the chalcone concentration increased, so did the antioxidant activity. In comparison to the standard l-ascorbic acid, which has an IC_50_ value of 15.06 µg/mL, the DPPH^**∙**^ IC_50_ values of chemosensors **1** and** 2** were determined as 25.47 ± 0.15 and 50 ± 0.19 µg/mL, respectively. Additionally, the IC_50_ values of chemosensors **1** and **2** for ABTS^+^ were estimated as 28.75 ± 0.25 and 42.75 ± 0.1 µg/mL, respectively, as opposed to the IC_50_ value for standard l-ascorbic acid, which is 8.368 µg/mL. This scavenging effect was brought about by the inclusion of the dihydropyridine ring, which can oxidize and lose a proton with the assistance of the electron-donating effects of the dimethyl-4-vinyl benzenamine fragment in chemosensor **1** and the 4-but-1,3-dienyl-diamethyl benzenamine fragment in chemosensor **2**, which reduce to the DPPH^**∙**^ and ABTS^+^ free radicals (Fig. 17)^41^. The difference in chain length between chemosensors **1** and **2** may account for chemosensor **1**’s greater ability to quench free radicals utilizing both DPPH^**∙**^ and ABTS^+^ scavenging tests than chemosensor **2**, which demonstrated a moderate impact.

### Molecular docking design and ADMET pharmacokinetics studies

**Figure 18:**
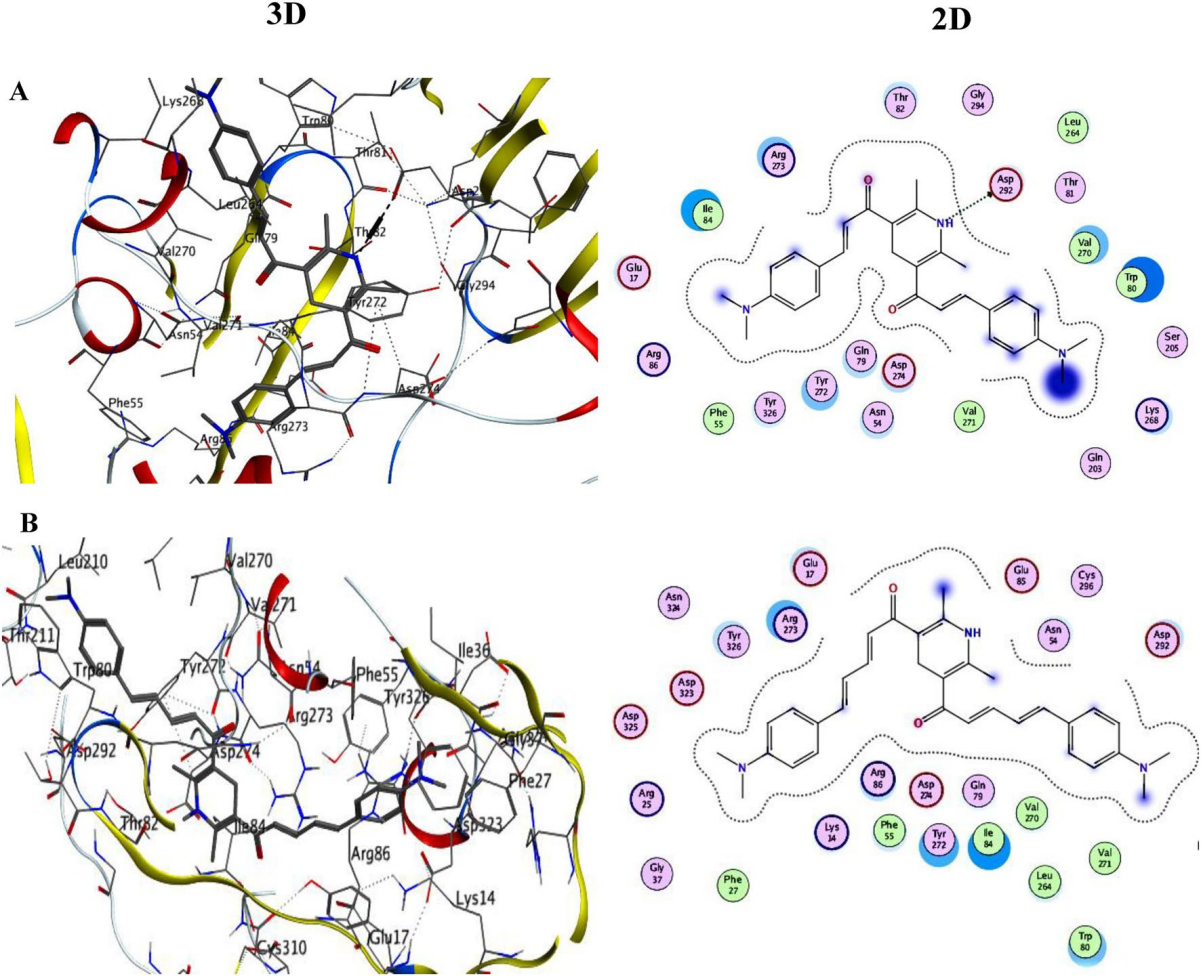
The interaction 2D–3D between (**A**) chemosensor **1** and (**B**) chemosensor **2** with the AkT target protein.

Docking experiments were carried out between the newly synthesized chemosensor molecules and the target protein to explore new Akt drug candidates^42^. The two screened chemosensors **1** and **2** have docking scores of − 11.9 and − 8.9 kcal/mol against the Akt target protein, respectively. The binding energy of Chemosensor **1** to the Akt protein is the greatest. Through H-bonds and –cation interactions with the amino acid residues Ile84, Arg273, Thr82, Asp292, Leu264, Val270, Trp80, Lys268, Gln79, Asn54, Asp274, Tyr272, Tyr326 and Glu17, respectively, Chemosensor **1** docked to the protein AkT. While the amino acid residues Arg273, Tyr326, Asp292, Asn54, Tyr272, Glu17, Ile84, Arg86, and Trp80 of chemosensor **2** interacted with the protein Akt, respectively (Table 4). The intermolecular interactions between the docked chemosensors and the target protein are shown in Fig. 18 in both 2D and 3D.

**Table 4:** Calculated docking scores (kcal/mol) of the docked chemosensors with the target protein.

Chemosensors	AkT (target protein)		
	Docking score (ΔG bind)	Docked complex (amino acid–ligand) interaction	Distance (Å)
1	− 11.9	Arg273	4.48
		Asp292	2.87
		Leu264	3.52
		Val270	3.3
		Lys268	2.63
		Asp274	3.47
		Tyr272	3.14
		Tyr326	2.06
		Glu17	2.34
		Ile84	2.01
		Asn54	3.23
		Trp80	2.12
		Gln79	2.25
		Thr82	3.26
2	− 8.9	Arg273	3.07
		Tyr326	2.49
		Asp292	3.09
		Asn54	4.2
		Tyr272	2.82
		Glu17	2.23
		Ile84	3.78
		Arg86	3.14
		Trp80	2.07

**Figure 19:**
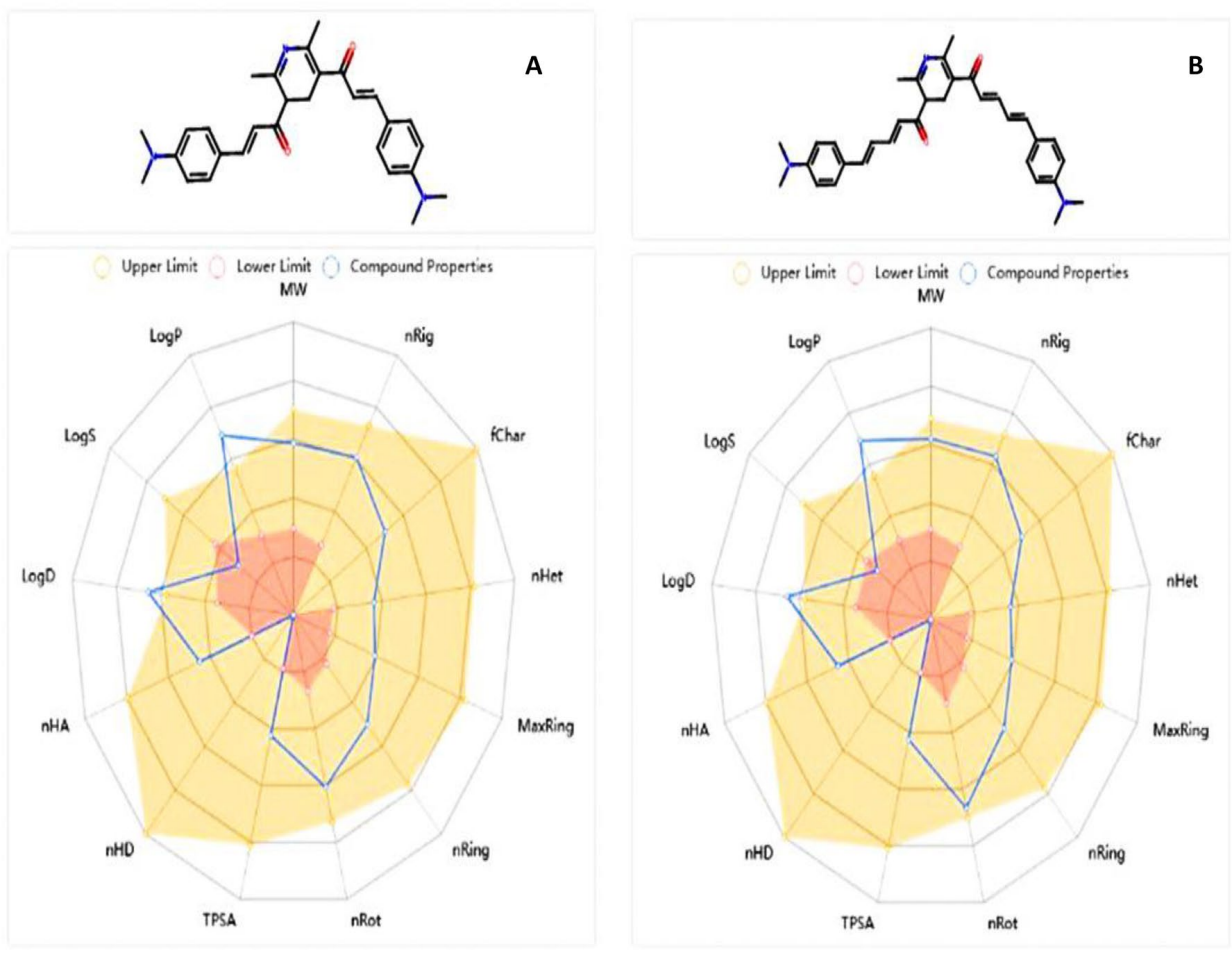
ADMET pharmacokinetics features for the docking interaction compounds as, (**A**) chemosensor **1**, (**B**) chemosensor **2**.

To ascertain the molecular and pharmacokinetic properties of the examined chemosensors **1** and **2**, see Fig. 19, ADMET is necessary. According to Lipinski's rule, the investigated chemosensors satisfied the Ro5 (no. of violations ≤ 1), satisfy all criteria for excellent permeability, and had a sufficient oral bioavailability as the total polar surface area (TPSA) of chemosensors **1** and **2** was between 52.65 and 52.980 Å_2_. Additionally, they demonstrated rotatable bonds with a number between 0 and 10, demonstrating flexibility. Their hydrogen bound accepted (HBA) and hydrogen bound donated (HBD) values were in the fulfilled range, which contributed to their improved solubility in cellular membranes. Table 5 shows that good lipophilicity features were indicated by octanol/water partition coefficient (log p) values below 5. Additionally, according to the ADMET criteria, the chemosensors had greater human intestinal absorption (% HIA) ratings, which indicated that the human intestinal could absorb them more efficiently. Since they do not penetrate the blood–brain barrier, the tested chemosensors **1** and **2** offer an excellent safety profile for the central nervous system (CNS). Lastly, all AMES toxicity and carcinogenicity test findings came back negative, proving their safety.

**Table 5:** ADMET properties of the synthesized chemosensors **1** and** 2**.

	Molecular weight (g/mol)	Blood–brain barrier (BBB)	% human intestinal absorption (HIA +)	TPSA A2	Log p	HBA	HBD	N rotatable	AMES toxicity	Carcinogenicity
Acceptable ranges	≤ 500	No	> 80% high < 30% low	≤ 140	< 5	2.0–20.0	0.0–6.0	≤ 10	Non toxic	Non carcinogenic
1	455.260	No	96.6	52.6	4.430	2	1	8	Non toxic	Non carcinogenic
2	507.678	No	98.38	52.9	4.624	2	1	10	Non toxic	Non carcinogenic

### Antitumor in-vitro study

**Figure 20:**
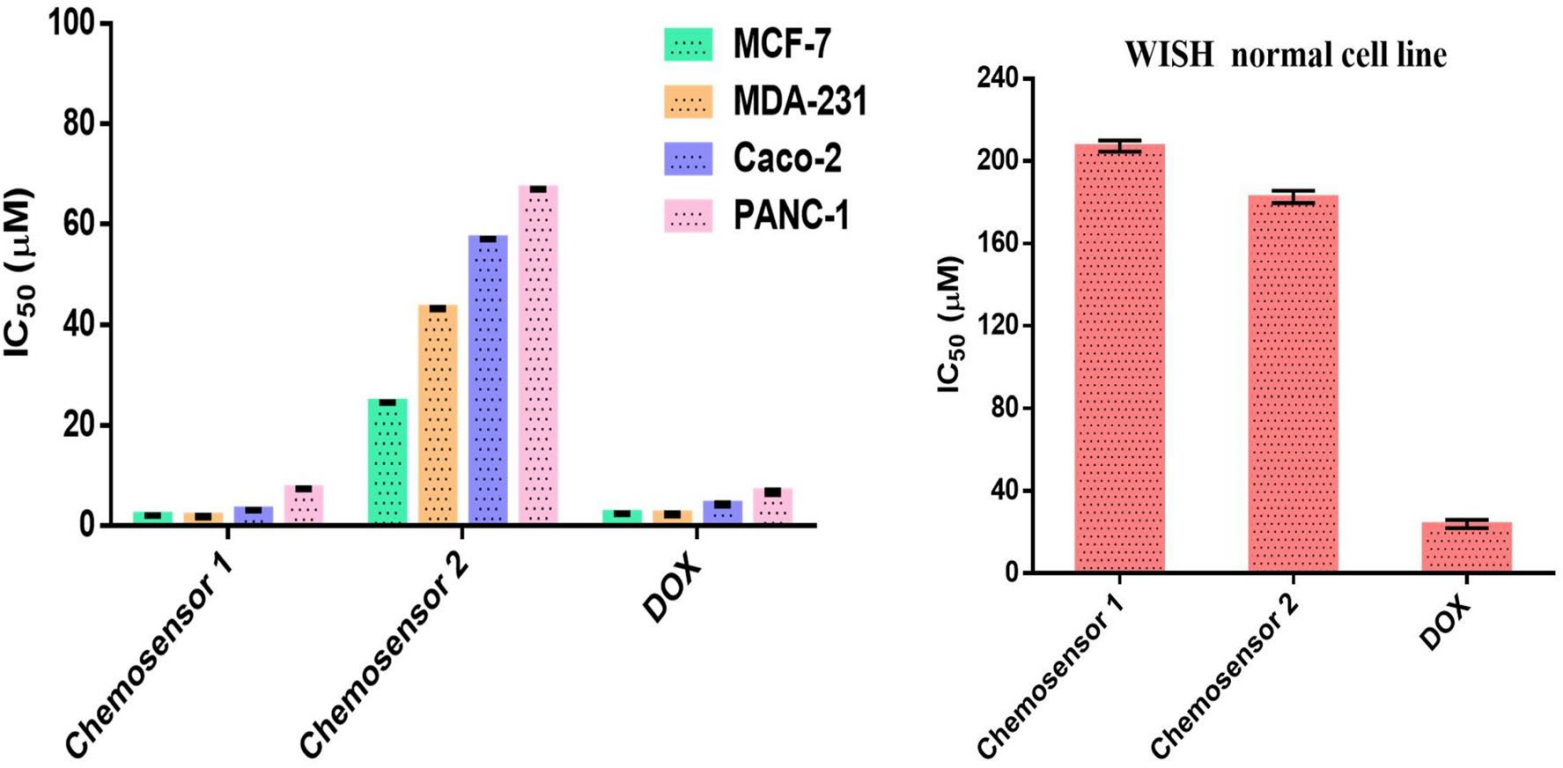
Chemosensors **1, 2** and doxorubicin inhibit the proliferation of different cancer cell lines. The IC_50_ values of each drug were expressed as mean ± SE.

Testing against a variety of different cancer cell lines is one of the most popular screening techniques used in studies on potential anticancer drugs^43^. The antitumor effect of chemosensors **1** and **2** on MDA-231, Caco-2, PANC-1 and MCF-7 proliferation, along with the cytotoxicity limit on WISH normal cell line after 48 h incubation, were assessed in this work using an MTT test (Fig. 20). Chemosensor **1** had substantial anticancer effects on the cell lines MCF-7, MDA-231, Caco-2, and PANC-1 cancer cell lines with an IC_50_ equal to 2.009 ± 0.14, 1.827 ± 0.29, 3.100 ± 0.14, and 7.346 ± 0.22 µM, respectively. While chemosensor **2** had a moderate cytotoxic impact on the cancer cell lines MCF-7, MDA-231, Caco-2, and PANC-1 cancer cell lines with an IC_50_ values of 24.52 ± 0.19, 43.17 ± 0.35, 57.06 ± 0.29, and 67.23 ± 0.32 µM, respectively. Additionally, WISH normal cells exposed to chemosensors 1 and 2 had less cytotoxic effects (IC_50_ = 207.3 ± 2.8 and 182.6 ± 2.7 µM), respectively. According to our findings, chemosensor **1** not only shown a more pronounced antiproliferative impact than chemosensor **2** against a panel of cancer cells, but it also had no harmful effects on healthy cells. Chemosensor **1** further has the same potent anticancer effects as DOX, but without any restrictions on harmful effects on normal cells. Chemosensor **1**’s better performance is a result of the Akt target protein's significant capability for blockage. As a result, it can be used as a strong therapeutic candidate for the treatment of cancer.

**Figure 21:**
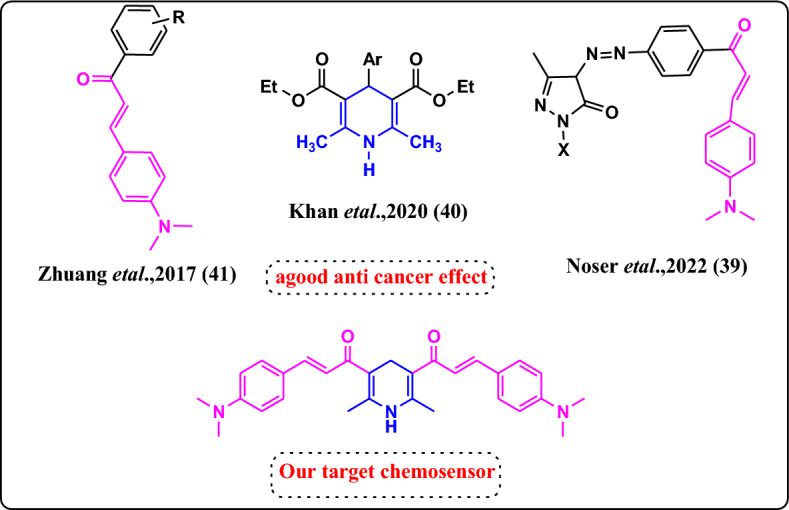
The structure activity relationship of chemosensors **1** anticancer impact.

The new synthesized chemosensors’ in-vitro anticancer and molecular docking studies showed the following SAR (Fig. 21):

- The presence of NH in dihydropyridine ring and carbonyl group enhanced the antitumor activity^44^ due to their ability to make H-bonding with the target protein.
- The aromatic rings enhanced the antitumor activity through π-π interaction^42,45^ with the active site of the target protein.
- The chain length between the dihydropyridine ring and the electron withdrawing CH_3_ groups is inversely related to the anticancer activity, and the presence of electron donating groups increased the anti-cancer impact^42,45^.

## Conclusion

In this study, we have successfully developed novel chemosensors for rapid colorimetric detection of different metal ions with high selectivity and sensitivity. Their structural characterizations were confirmed by FT-IR, ^1^H-NMR, ^13^C-NMR, elemental analysis and UV–Visible measuremnets. The solvatochromic behavior was explored in different solvents of various polarities to explore the active fluorescent tautomer. The visual detection, as well as UV–Vis and fluorescence measurements were carried out to explore the colorimetric and optical sensing properties of the investigated chemosensors toward various metal ions such as Al^3+^, Cr^3+^, Mn^2+^, Fe^3+^, Co^2+^, Ni^2+^, Cu^2+^, Mg^2+^, Hg^2+^ and Zn^2+^. Chemosensors selectively detected metal ions through a color change. The chemosensors were totally reversible by addition of EDTA to the formed complexes. The chemosensors displayed turn on–off-on fluorescence response based on an effect of chelation-quenching fluorescence. The obtained results confirming the powerful sensing ability of chemosensors with high sensitivity and selectivity toward some of the studied metal ions. Also, the chemosensors were highly promising for on-site and real-time colorimetric monitoring of metal ions in environmental and biological samples. Finally, these chemosensors showed highly antioxidant, anticancer impact and this was in accordance with their in-silico binding energies against Akt target proteins also, they obey Lipinski rule of 5.

### Supplementary Information

Supplementary Figures.

## Data Availability

The datasets generated and/or analysed during the current study are available in: Macromolecule protein structure, can be deposited in the worldwide protein data bank repository, (https://www.rcsb.org/structure/4EJN). All cell lines were purchased from the American Type Culture Collection (ATCC) organization (#ATCC HTB-22, #ATCC HTB-26, #ATCC HTB-37, #ATCC CRL-1469 and #ATCC CCL-25).
