# Supplementary material for: Simple dihydropyridine-based colorimetric chemosensors for heavy metal ion detection, biological evaluation, molecular docking, and ADMET profiling
Source: Sci Rep. 2023 Sep 18;13:15420. doi: 10.1038/s41598-023-42137-7 (PMC10507071; doi:10.1038/s41598-023-42137-7)
Supplement: Supplementary file 1 — Supplementary Figures. [file 41598_2023_42137_MOESM1_ESM.pdf]

<u>**Supplementary material**</u>

Simple dihydropyridine-based colorimetric chemosensors for heavy metal ion detection, biological evaluation, molecular docking, and ADMET profiling.

Wafaa M. Hamada[1], Marwa N. El-Nahass[1]*, Ahmed A Noser[1], Tarek A. Fayed[1], Maged El-Kemary[2], Maha M. Salem[3], Eman A. Bakr[1]

[1]Chemistry Department, Faculty of Science, Tanta University, 31527 Tanta, Egypt
[2]Institute of Nanoscience and Nanotechnology, Kafrelsheikh University, Kafr El-Sheikh33516, Egypt
[3]Biochemistry Division, Chemistry Department, Faculty of Science, Tanta University, 31527 Tanta, Egypt

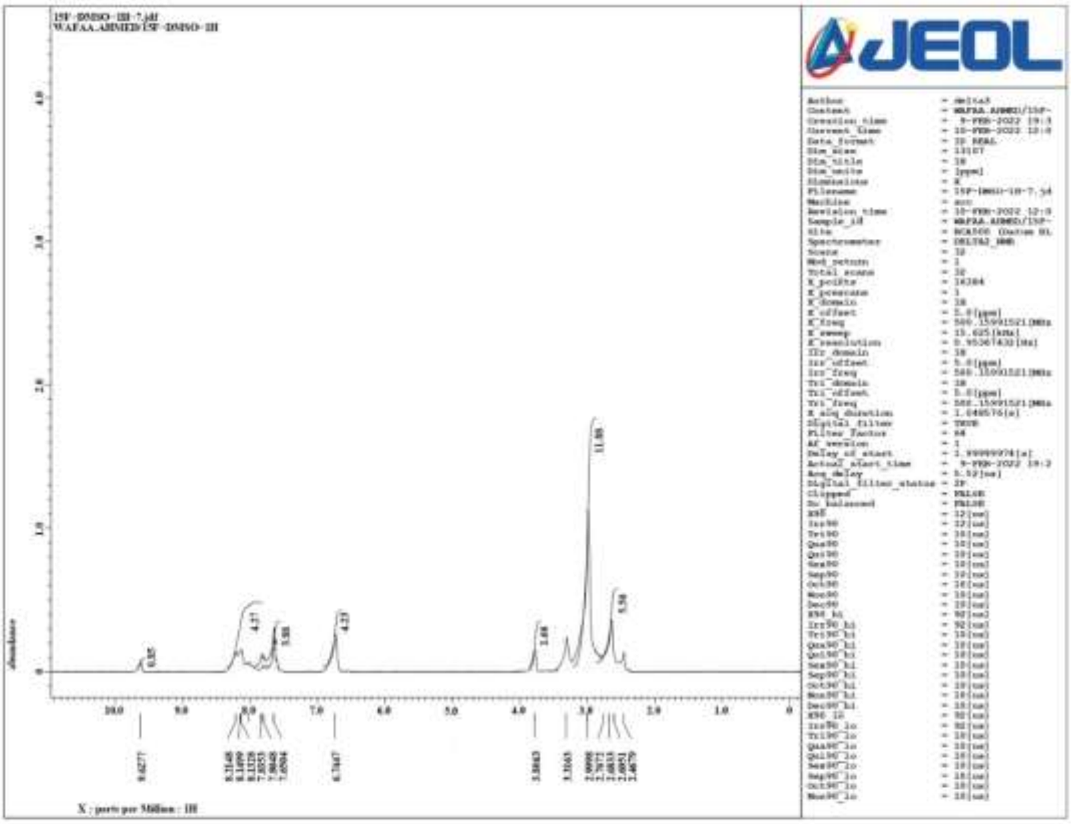

Fig S1: The original data of ^{1}H NMR spectra of chemosensors **1**

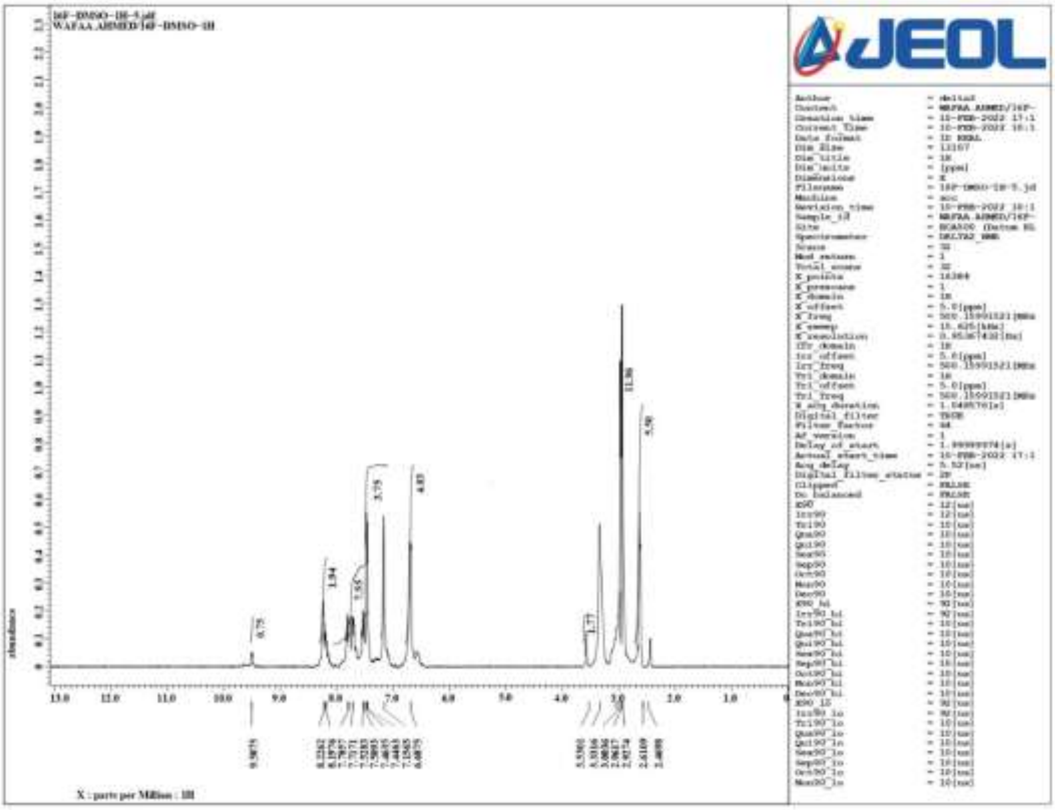

Fig S2: The original data of ^{1}H NMR spectra of chemosensors **2**

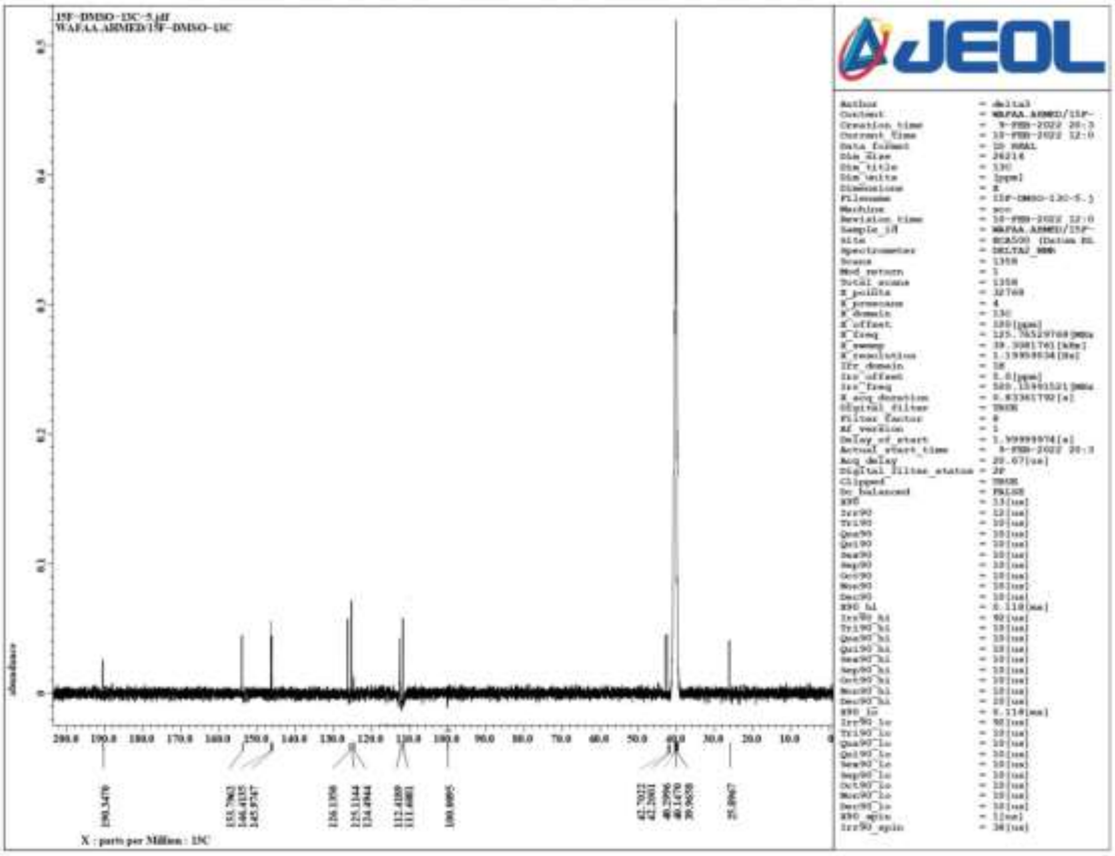

Fig S3: The original data of ^{13}C NMR spectra of chemosensors **1**

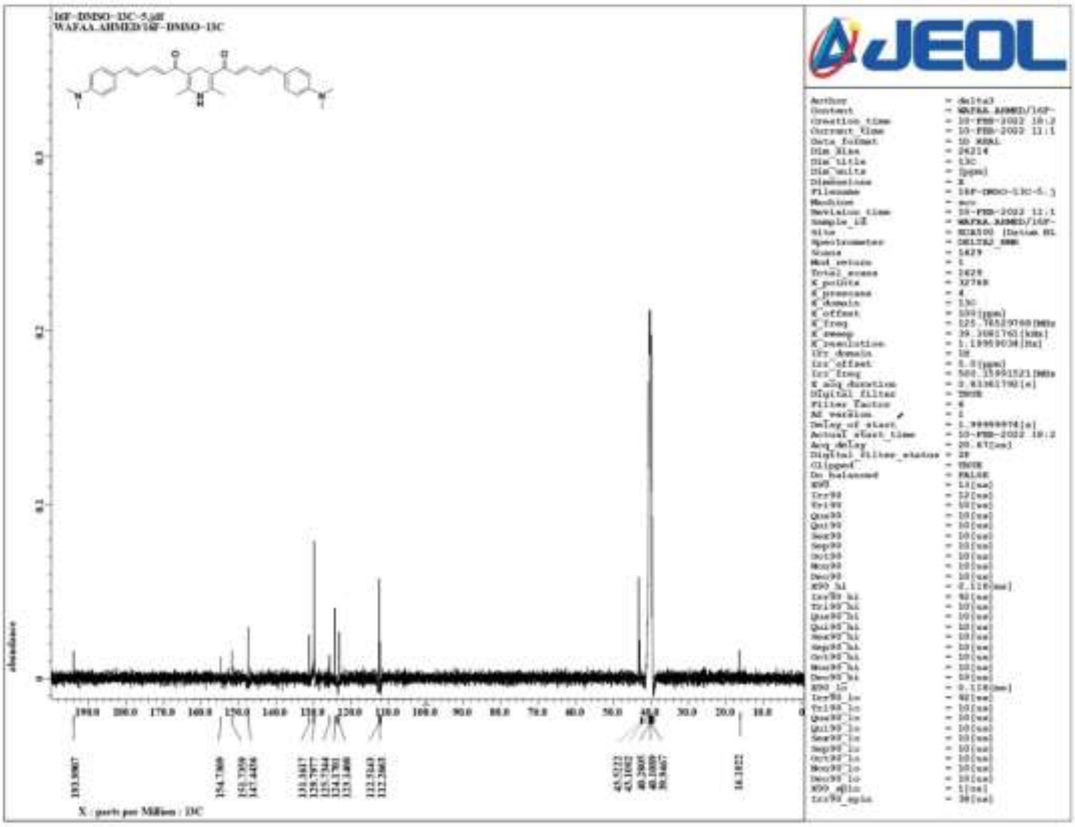

Fig S4: The original data of ^{13}C NMR spectra of chemosensors **2**

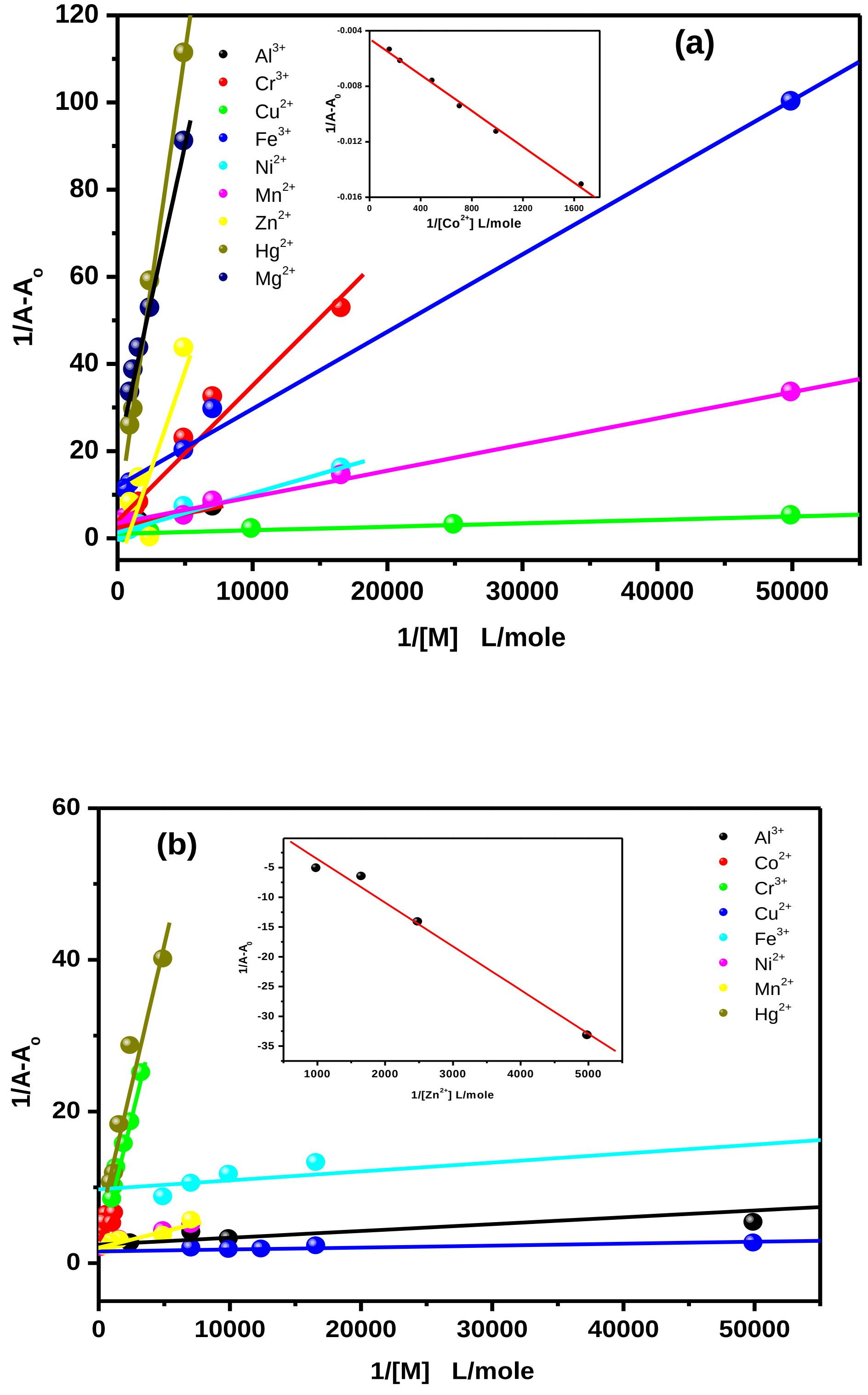

Fig.S5. Benesi-Hildebrand plots for the binding of the chemosensors (a) 1 and (b) 2 with different metal ions.

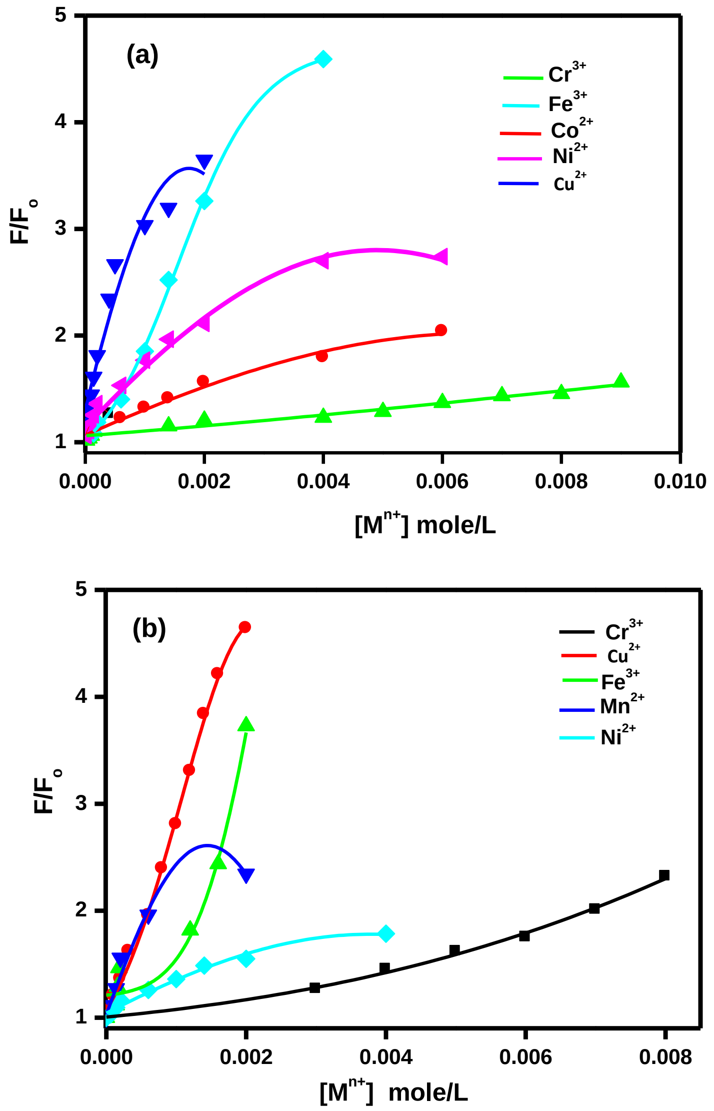

Fig.S6. Stern–Volmer plots for the quenching of the chemosensors (a) 1 and (b) 2 by different metal ions.
